# Thyroid Disorders in Systemic Sclerosis: A Comprehensive Review

**DOI:** 10.3390/jcm13020415

**Published:** 2024-01-11

**Authors:** Aifer Cherim, Răzvan-Cosmin Petca, Mihai-Cristian Dumitrascu, Aida Petca, Elisabeta Candrea, Florica Sandru

**Affiliations:** 1 Department of Dermatovenerology, ‘Carol Davila’ University of Medicine and Pharmacy, 050474 Bucharest, Romania; aifer.cherim@rez.umfcd.ro (A.C.); florica.sandru@umfcd.ro (F.S.); 2 Internal Medicine 3rd Department, Colentina Clinical Hospital, 020123 Bucharest, Romania; 3 Department of Urology, ‘Carol Davila’ University of Medicine and Pharmacy, 050474 Bucharest, Romania; razvan.petca@umfcd.ro; 4 Department of Urology, ‘Prof. Dr. Th. Burghele’ Clinical Hospital, 050659 Bucharest, Romania; 5 Department of Obstetrics and Gynecology, ‘Carol Davila’ University of Medicine and Pharmacy, 050474 Bucharest, Romania; mihai.dumitrascu@umfcd.ro; 6 Department of Obstetrics and Gynecology, University Emergency Hospital of Bucharest, 050098 Bucharest, Romania; 7 Department of Obstetrics and Gynecology, Elias Emergency University Hospital, 011461 Bucharest, Romania; 8 Department of Dermatology, University of Medicine and Pharmacy ‘I. Hatieganu’, 400347 Cluj Napoca, Romania; elisabeta.candrea@umfcluj.ro; 9 Dermatology Department, “Elias” University Emergency Hospital, 011461 Bucharest, Romania

**Keywords:** systemic sclerosis, thyroid disorders, thyroid cancer, familial autoimmunity

## Abstract

Systemic sclerosis, also referred to as scleroderma, is a chronic autoimmune disease that affects both internal organs and the skin. Systemic sclerosis predominantly affects female patients and can coexist with other disorders, including those affecting the thyroid gland. Common symptoms such as fatigue and weight changes can be attributed to either systemic sclerosis or thyroid disease. In this comprehensive review, an extensive analysis is conducted using research from 2002 to 2022, sourced from PubMed. The main focus of this exploration is to understand the intricate relationship between thyroid disorders and systemic sclerosis. We obtained these results by analyzing a number of 32285 patients included in 21 original studies. The existing evidence suggests that there is a higher incidence of elevated TSH levels and hypothyroidism in patients with systemic sclerosis, particularly in females, compared to the general population. This remains true even when comparing patients from iodine-deficient regions. Additionally, there is an increased occurrence of hyperthyroidism in the context of systemic sclerosis, which negatively impacts the prognosis of these patients. Furthermore, thyroid antibodies, predominantly anti-thyroid peroxidase (anti-TPO) antibodies, and autoimmune disorders are more commonly observed in individuals with systemic sclerosis. Although thyroid nodules are not specifically linked to the disease, when considering thyroid volume, it is observed that the thyroid gland in systemic sclerosis patients has a decreased volume, possibly due to fibrosis. Conversely, other studies have revealed that patients without autoimmune thyroid diseases (AITDs) are more likely to have a history of digital ulcers, pulmonary fibrosis detected by computed tomography scan, and a requirement for immunosuppressive medication. The majority of the studies did not establish a connection between thyroid disease in these patients and the occurrence of the limited or diffuse forms of systemic sclerosis, as well as the presence of digital ulcers, calcinosis, pulmonary arterial hypertension, scleroderma renal crisis, Raynaud phenomenon, and various other clinical manifestations.

## 1. Introduction

### 1.1. Systemic Sclerosis

Systemic sclerosis (SSc), or scleroderma, is a chronic, heterogeneous, multisystemic autoimmune disease affecting multiple internal organs and the skin [1]. Vascular damage, immunological abnormality, and fibrosis are the three manifestations that define the mechanism behind SSc. It has the highest mortality rate of all rheumatic diseases [2,3]. The characteristic feature of this disease is the thickening of the skin, also known as scleroderma. Raynaud’s phenomenon (RP) is observed in nearly all individuals diagnosed with SSc [4], commonly serving as an early indication of the disease, along with the presence of fatigue [4,5]. SSc is classified according to the extent of skin involvement and the accompanying pattern of internal organ involvement: limited cutaneous SSc (lcSSc), diffuse cutaneous SSc (dcSSc), SSc without scleroderma, and SSc with overlap syndrome [6]. The term lcSSc denotes the manifestation of skin thickening occurring distally to the elbows and knees, with or without the involvement of the face. Conversely, dcSSc is characterized by skin thickening that extends proximally and distally to the elbows and knees, with or without affecting the face [7].

The different subtypes of the disease are also associated with varying patterns of organ involvement and disease progression. Patients with SSc may experience life-threatening internal organ involvement, including interstitial lung disease (ILD), pulmonary arterial hypertension (PAH), heart failure, severe gastrointestinal tract (GIT) involvement, and scleroderma renal crisis (SRC) [8,9,10,11]. Physicians utilize the modified Rodnan skin score (mRSS) to evaluate the progression of cutaneous fibrosis over time. A deteriorating mRSS is linked to increased mortality rates, as well as negative impacts on renal and cardiac health outcomes [12]. The diagnostic standards for SSc have changed dramatically over time due to the multifaceted nature of the disease’s pathophysiology and symptoms. The American College of Rheumatology (ACR) 1980 criteria were first proposed by the ARA in 1980 as preliminary standards for classifying SSc. These were replaced by a new set that LeRoy proposed in 1988 [6]. LeRoy and Medsger revised these criteria in 2001 and separated the disease into three subsets: diffuse cutaneous, limited cutaneous, and limited SSc [13]. New classification criteria, developed in 2013 due to cooperation between the ACR and the European Alliance of Associations for Rheumatology (EULAR), outperformed the 1980 ACR criteria in terms of sensitivity and specificity [1].

The diagnosis of SSc is primarily based on clinical findings and supported by laboratory criteria [14]. SSc is a disease characterized by the presence of autoantibodies. The three main types of autoantibodies that have been identified in more than 90% of the SSc patients are anti-topoisomerase I antibodies (ATAs), also known as anti-Scl70 antibodies, anti-RNA polymerase III antibodies (ARAs), and anti-centromere antibodies (ACAs) [1,15]. Moreover, research revealed that ACAs, ATAs, and ARAs have pathological functions beyond disease diagnosis [16,17]. ACAs are usually found in patients with limited cutaneous SSc [16] and are often associated with PAH [18]. ATAs are typically found in patients with diffuse SSc and are linked to ILD [19,20], higher SSc-related mortality rates, and poor prognosis [21]. ARAs are most associated with SRC [21].

The disease presents with varying degrees of severity and prognosis, ranging from mild manifestations to rapidly progressive renal failure. The choice of treatment regimens primarily depends on the specific organs impacted by the disease [22]. Certain patients may only require medication for clinical manifestations, such as RP, while others may necessitate intensive immunosuppressive therapy.

Autoimmune disorders, such as SSc, exhibit a heightened occurrence rate of thyroid disorders; however, there is limited knowledge regarding the clinical manifestations of SSc in patients who concurrently present with thyroid disease.

### 1.2. Hypothyroidism

Hypothyroidism arises either from insufficient thyroid hormone production or the insufficient stimulation of the thyroid gland by the hypothalamus or pituitary gland, the causes varying from primary gland failure to iatrogenic, transient, or central [23,24,25,26]. Iodine deficiency is the most common cause of primary hypothyroidism in iodine-deficient regions of the world. In the iodine-sufficient areas and the United States, autoimmune thyroid diseases (AITDs) are the primary cause of hypothyroidism, with Hashimoto’s thyroiditis (HT) being the most frequent cause of hypothyroidism in the United States. In most patients, screening for primary hypothyroidism is performed using the serum thyroid-stimulating hormone (TSH) and free thyroxine (FT4) levels. Low levels of FT4 and high TSH are characteristic of overt hypothyroidism, while TSH is elevated and FT4 is normal in subclinical hypothyroidism [27]. Anti-thyroid antibodies, such as anti-thyroid peroxidase (anti-TPO) antibodies, should be measured as part of the laboratory work for AITDs. Anti-TPO antibody-positive patients with subclinical hypothyroidism are more likely to develop overt hypothyroidism [24]. Clinical hypothyroidism is more frequent in people over 65 years old [27,28,29]. It is seven times more common in females than males (40 out of 10,000 vs. 6 out of 10,000) [30]. Like most autoimmune diseases, SSc is also more commonly found in female patients [31].

### 1.3. Hyperthyroidism

On the other hand, an excessive production of thyroid hormone is the defining characteristic of hyperthyroidism [32]. This condition can manifest as overt or subclinical. Overt hyperthyroidism is diagnosed when TSH levels are low or suppressed, accompanied by elevated levels of triiodothyronine (T3) and/or thyroxine (T4) [32]. The condition of subclinical hyperthyroidism is marked by a decrease in or suppression of TSH levels, while T3 and T4 levels remain unaltered [33]. Hyperthyroidism can be attributed to three primary factors, namely, Graves’ disease (GD) [34], toxic multinodular goiter, and toxic adenoma. The most prevalent cause of hyperthyroidism in the United States and other western nations is GD [34]. Due to its etiology, this autoimmune disorder is more commonly observed in younger populations. However, in regions with iodine deficiency among older adults, toxic multinodular goiter is the most common cause of hyperthyroidism [35,36,37]. The presence of hyperthyroidism is linked to both immediate and long-term health complications.

### 1.4. Autoimmune Thyroid Diseases

It is estimated that the combined prevalence of HT and GD is around 5% in the general population [28,38,39], while the prevalence of anti-thyroid antibodies without any clinical disorder may be higher [40]. The etiology is complex and involves a combination of genetic and environmental factors such as infections, dietary factors, iodine intake, and smoking [41].

In HT, the major autoantigens are thyroid peroxidase (TPO) and thyroglobulin (Tg), while in GD, the major autoantigen is the thyroid-stimulating hormone receptor (TSHR) [42]. However, TSHR autoantibodies can also occur in a subset of patients with HT.

### 1.5. Thyroid Cancer

Several studies have provided evidence suggesting that patients diagnosed with SSc exhibit a heightened susceptibility to the development of cancer. The emergence of diverse cancer types in correlation with SSc or SSc-like conditions indicates a variety of underlying mechanisms, consisting of modified immune responses, shared genetics, environmental factors, disease-dependent variables, and biologic substances derived from tumors and therapies [43]. However, the specific risk associated with the development of thyroid cancer in these individuals remains relatively unknown, with isolated cases of papillary thyroid cancer being described in the literature [44,45,46,47]. The incidence of thyroid cancer in the general population is increasing over time; approximately 1% of all malignancies are attributed to thyroid carcinoma, according to estimates. This form of cancer encompasses a diverse array of variants, each characterized by distinct clinical behaviors, patterns of progression, and prognostic outcomes. These variants include papillary, medullary, follicular, and Hurthle cell thyroid cancer, collectively known as differentiated thyroid cancer. Undifferentiated forms of cancer may also manifest within the thyroid gland, typically associated with a more unfavorable prognosis. In countries with sufficient iodine diets, PTC is the most observed malignant thyroid neoplasm, accounting for up to 80% of all thyroid malignancies. While PTC can affect individuals of all age groups, it is most frequently diagnosed in the 3rd to 5th decades of life. On the other hand, follicular thyroid carcinoma (FTC) is more prevalent in regions with inadequate iodine diets and constitutes approximately 10 to 20% of all thyroid malignancies. FTC can occur across a wide range of ages but is most commonly diagnosed in individuals in their 5th and 6th decades [48,49]. Undifferentiated thyroid carcinoma (UTC), which accounts for up to 10% of cases, typically manifests in patients beyond the age of 60. 

### 1.6. Thyroid Nodules

The thyroid nodule, as described by the American Thyroid Association, is a discrete lesion within the thyroid gland that can be distinguished radiologically from the surrounding thyroid parenchyma [50]. Thyroid nodules are a commonly encountered condition, with physical examinations alone detecting them in approximately 5% to 7% of the adult population. Nodules are also more common in females and older age groups; less than 25% of thyroid nodules reach >1 cm [51]. These nodules can exist as solitary entities or occur in multiple numbers, and can either be cystic or solid [52]. While a significant proportion of detected nodules are benign and medically insignificant [53], it is essential to acknowledge the clinical relevance of thyroid nodules, as they can potentially indicate thyroid cancer in approximately 4.0% to 6.5% of cases [54]. The occurrence of multiple thyroid nodules or a multinodular goiter does not contribute to an increased susceptibility to thyroid cancer. It is recommended to evaluate each nodule independently, focusing on identifying any high-risk sonographic features that may indicate the presence of malignancy [50]. In certain instances, thyroid nodules can result in an overproduction of thyroid hormones, although it is exceedingly rare for these nodules to be cancerous [55].

The need for focused attention on the potential relationship between thyroid disorders and SSc is essential, as it plays a critical role in guiding the decision-making process for preventive, screening, and therapeutic approaches toward thyroid diseases in SSc patients. Numerous studies have demonstrated an increased occurrence of thyroid disorders among individuals with SSc, although there exists a divergence of findings on this subject. This article aims to comprehensively analyze the literature spanning the past two decades, focusing on the prevalence of thyroid gland disorders in SSc patients.

## 2. Materials and Methods

We reviewed English papers on PubMed covering 20 years of recent publications (from 2022 to 2002) using different combinations of keywords, such as “SSc” and “thyroid disease” or “scleroderma”, “hypothyroidism”, “hyperthyroidism, “thyroid cancer”, “Hashimoto’s disease”, “Graves’ disease”, “autoimmune thyroid disorders”, “thyroid volume”, “thyroid nodules”, and “familial autoimmunity”.

We excluded case reports or case series from our survey.

**Figure 1:**
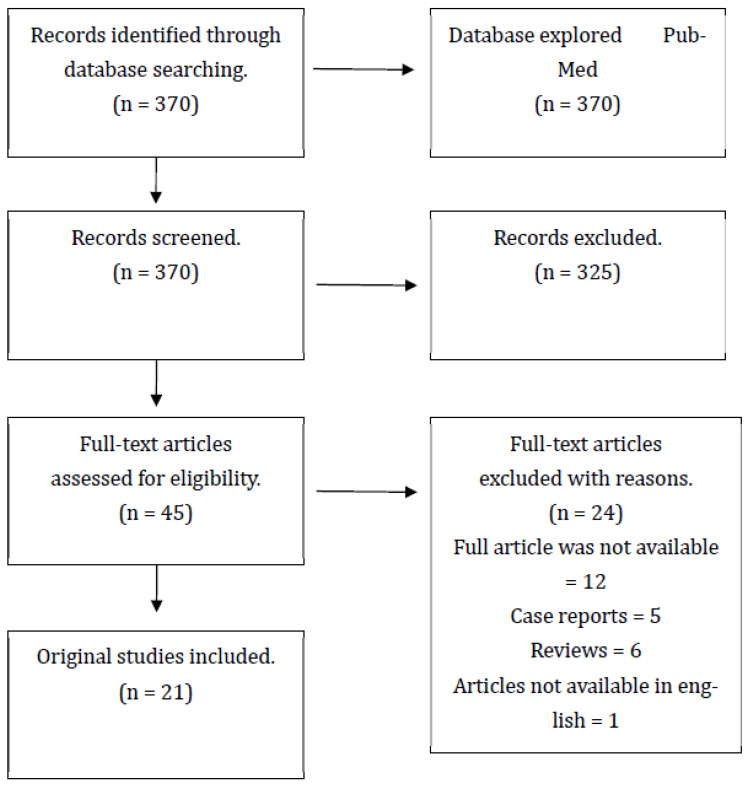
Literature selection and data extraction.

A total of 370 studies were identified. All resulting studies were screened based on title and abstract, and 325 were excluded. The majority of the excluded studies did not fall into the targeted theme or targeted population. After full-text screening, the 21 most relevant articles were selected to form the final review—Figure 1.

We classified the findings into four categories: SSc and thyroid dysfunction, thyroid autoimmune disease, and thyroid cancer; SSc and thyroid volume and nodules; clinical manifestations in SSc patients with thyroid disorders; and SSc, thyroid autoimmune disorders, and familial autoimmunity.

## 3. Results 

### 3.1. Thyroid Dysfunction, Autoimmunity, and Cancer in SSc

In a cross-sectional study by Akuka A et al. in 2019 involving 15,141 participants, comprising 2431 individuals diagnosed with SSc and 12,710 controls, a higher prevalence of hyperthyroidism among SSc patients was observed, exhibiting statistical significance. Furthermore, all-cause mortality was significantly higher in SSc patients with hyperthyroidism (37.8%) compared to those without (25.8%), control patients with (22.1%), and without hyperthyroidism (12.3%). It is important to highlight that individuals with both SSc and hyperthyroidism were predominantly females, underweight, and had a lower socioeconomic status [56].

In the context of another cross-sectional investigation involving 790 participants, which included 649 individuals diagnosed with autoimmune diseases and 141 designated as controls, the findings revealed a prevalence of 27.5% for hypothyroidism among the subset of 58 individuals diagnosed with SSc. In comparison, only 11.3% of the control patients exhibited this condition. The statistical significance of these findings, however, was not established. Additionally, this study found a higher prevalence of anti-thyroid antibodies, either with or without hypothyroidism, when compared to controls [57].

Antonelli A and colleagues analyzed 1635 patients, 327 with SSc, 654 controls from iodine-deficient areas, and 654 controls from iodine-sufficient areas. They observed that patients with SSc exhibited higher levels of TSH, more cases of hypothyroidism (both clinical and subclinical), and higher levels and prevalence of anti-TPO and anti-thyroglobulin (anti-Tg) antibodies in comparison to control patients, the difference reaching statistical significance. Subsequently, the study found that individuals with SSc had lower free triiodothyronine (FT3) and FT4 levels than controls. However, the difference did not reach statistical significance. No disparity was observed in the incidence of clinical or subclinical hyperthyroidism across the three groups [58]. Furthermore, the study demonstrated the occurrence of papillary thyroid cancer in six patients with SSc, indicating a statistically significant difference when compared to the control group. In terms of the comparison between SSc patients with and without thyroid cancer, various factors were analyzed, including age, the distribution of sexes, the type of SSc, and the presence of antibodies such as anti-nuclear antibodies (ANAs), ACAs, ATAs, anti-Tg, and anti-TPO. No statistically significant differences were noted, except for the presence of anti-TPO antibodies, which were found to be more prevalent in SSc patients with papillary thyroid cancer. The theory proposed by the scientists was that the link between thyroid cancer and SSc could be attributed to the chronic inflammatory process caused by thyroiditis or the activation of the extracellular signal-regulated kinases (ERKs) pathway by a common proto-oncogene that is common to both conditions [58]. 

Continuing the discussion on thyroid cancer, in a study by Toki S et al., encompassing a sample of 210 SSc patients, 30 of whom also presented with AITDs, 29 with HT and 1 with GD, ultrasonography findings did not yield any statistically significant evidence supporting the presence of thyroid cancer in SSc patients with AITDs, in comparison to patients without AITDs [59]. 

In another study, conducted by Derk CT et al. and involving a cohort of 769 systemic sclerosis (SSc) patients, thyroid cancer was observed in just 2 individuals after the SSc diagnosis. However, the limited number of cases precluded reaching statistical significance [60].

A more recent 2022 study, involving a total of 8120 patients diagnosed with autoimmune diseases, of which 375 presented with SSc, revealed that 7.2% of the SSc patients were afflicted by cancer. The standard incidence ratio was calculated to be 3.77 (95% CI = 2.49–5.49), with the highest standard incidence ratio for cervical cancer, followed by lung and breast cancer. Unfortunately, the standard incidence ratio for thyroid cancer could not be established through this study, as no cases of thyroid cancer were observed among these patients [61].

Recentering the focus on thyroid dysfunction, in a longitudinal study carried out by Antonelli A et al., which involved 358 female participants, 179 patients with SSc and 179 controls who were monitored for durations of 73 and 94 months, respectively, no discernible disparities in thyroid hormones were observed during the initial presentation; however, the levels of anti-TPO and the percentage of patients with higher anti-TPO levels were higher and statistically significant in the SSc group compared to the control group. A more significant number of patients in the SSc group showed elevated levels of anti-Tg antibody compared to the control group but failed to reach statistical significance. No case of GD was initially detected. 

However, during the final assessment, it was observed that patients with SSc exhibited elevated levels of TSH, subclinical hypothyroidism, and a higher incidence of thyroid dysfunction compared to controls, with statistical significance. Furthermore, the prevalence of clinical hypothyroidism and subclinical hyperthyroidism was similar in SSc patients compared to control subjects [62]. The levels of anti-TPO and the percentage of patients with elevated anti-TPO levels remained consistently higher in the SSc group compared to the control group, reaching statistical significance. However, the levels of anti-Tg antibodies, despite being higher in SSc patients compared to the control group, did not reach statistical significance. While GD was noted in two SSc patients, the observed cases did not show statistical significance when compared to the control group. Nonetheless, thyroid autoimmunity was more prevalent in the SSc group, and this difference reached statistical significance [62]. 

At the end of the follow-up, hypothyroidism was significantly associated with the presence of anti-TPO antibody positivity, a small thyroid volume, and a hypoechoic pattern [62].

Furthermore, no cases of thyroid cancer were initially observed among any of the patients included in the study. However, during the final evaluation, it was found that 1.1% of patients in the SSc group developed thyroid cancer, as opposed to zero cases in the control group. Nevertheless, the observed difference did not reach statistical significance [62].

In another investigation by Antonelli A and colleagues, which included 606 patients (202 SSc and 404 controls), subclinical hypothyroidism was present in 17% of female patients with SSc and 6% of male patients with SSc, in comparison to 6% of control female patients and 0 control male patients, respectively. This difference was statistically significant in the female patient group. TSH levels were significantly higher in female SSc patients when compared to controls. Clinical hypothyroidism was also more frequent in female patients with SSc (4%) compared to female controls (0.3%), with the difference reaching statistical significance The study further revealed that subclinical hyperthyroidism was observed more frequently in the female control group (6%) compared to female patients with SSc without reaching statistical significance [63]. Researchers found that female patients with SSc had slightly higher levels of anti-Tg antibodies than female control patients, and a higher number of patients presenting with anti-Tg antibodies. However, the difference was not statistically significant. On the other hand, the levels and prevalence of anti-TPO antibodies in female SSc patients were higher compared to controls. GD was also found to be significantly more prevalent in female SSc patients compared to female controls. A considerably higher percentage of female SSc patients presented with thyroid autoimmunity compared to control females. The same trend was observed in male patients, where anti-TPO antibody levels, the percentage of patients presenting with anti-TPO antibodies, and thyroid autoimmunity were significantly higher in male SSc patients than in male controls. In male patients, no statistically significant disparities were observed between those with systemic sclerosis (SSc) and the control group when assessing the levels of TSH, FT3, FT4, anti-Tg antibodies, subclinical hypothyroidism, and subclinical hyperthyroidism [64].

In a study by Shahin AA et al. involving a sample size of 38 individuals (24 with SSc and 15 controls), hypothyroidism was observed in 33.3% of the SSc patients. Furthermore, it was noted that the levels of FT4 were significantly lower in the SSc group compared to the control group. However, no significant difference was observed in the levels of FT3 between the SSc patients and the control group. It is worth mentioning that none of the patients exhibited hyperthyroidism in terms of FT4 levels [63].

In an investigation conducted by Meridor K et al. on a cohort of 50 individuals diagnosed with SSc, 10 patients had a pre-existing thyroid disease, 8 had autoimmune thyroiditis, and 2 underwent hemithyroidectomy for unknown reasons. A total of 17% of patients with SSc presented anti-thyroid antibodies. No statistically significant difference was observed in TSH and FT4 levels between SSc patients with no previously diagnosed thyroid disease when compared to SSc patients with thyroid disease [65].

Moving on to another study by Posselt RT and colleagues examining a cohort of 790 patients, among whom 649 individuals suffered from an autoimmune disease, including 58 with SSc, and 141 represented control subjects, anti-TPO antibodies associated with hypothyroidism were found to be more frequent in the SSc group (20.6%) compared to the control group (5.6%), the difference being statistically significant. In comparison to the control group, patients with scleroderma had a significantly higher likelihood of displaying anti-TPO antibodies with hypothyroidism (3.4-fold) and anti-TPO or/and anti-Tg without hypothyroidism (3.1 fold) [57].

Furthermore, a study by Wielosz E et al. involving 86 patients diagnosed with SSc, including 32 with diffuse SSc and 54 with limited cutaneous type, revealed that 59 did not present any thyroid autoimmune marker. The study showed that 27 (31%) patients in the SSc group had a positive anti-thyroid antibody, and AITDs were diagnosed in 26 patients with SSc. The prevalence of anti-thyroid antibodies (anti-TPO, anti-Tg, anti-TPO or anti-Tg, and anti-TPO and anti-Tg) showed no statistically significant difference between patients with lcSSc and dcSSc. Moreover, there was no significant difference in the occurrence of AITDs between the diffuse and the limited forms of SSc [66].

In a 2015 study by Bagnato et al. on a total of 105 female patients (70 SSc and 35 HT), anti-TPO and anti-Tg antibodies were found to be positive in more control patients with HT (62% and 51%, respectively) when compared to SSc patients both dcSSc and lcSSc (11% and 24%, respectively), with the difference being statistically significant. FT3 and FT4 levels were significantly lower, and TSH levels were significantly higher in female patients suffering from HT when compared to SSc patients. Seventeen (24%) SSc patients were found to suffer from HT [67].

A survey on 210 patients with SSc, 30 of whom also suffered from AITDs, 29 presenting HT and 1 GD, found that either or both anti-TPO and anti-Tg antibodies occurred in 28.6% of SSc patients. Half of these SSc patients also presented ultrasonographic findings. All of the SSc patients with AITDs were females. ANAs and ACAs were found more frequently in patients suffering from both SSc and AITDs, while ATAs were more frequent in SSc patients without AITDs, the difference being statistically significant [59].

A study by Avouac J et al. involving 1132 patients with SSc revealed that 6% also presented autoimmune thyroiditis **[68]**.

Furthermore, another study published in 2007, assessing the frequency of autoimmune thyroiditis in 118 patients with SSc, demonstrated that 17 patients (14.4%) had autoimmune thyroiditis. Two patients were diagnosed concurrently with autoimmune thyroiditis, eight patients were diagnosed after the SSc diagnosis, and seven patients were diagnosed before [69]. 

Lastly, in an investigation by Biro E. et al. involving 1517 patients with autoimmune diseases, 119 individuals were diagnosed with SSc. The study revealed that 4.2% of the SSc patients had HT, 3.4% had GD, and 4.2% presented euthyroid goiter. Overall, 7.6% of the SSc patients presented with an AITDs. Comparatively, when considering all patients with autoimmune diseases, the prevalence of AITDs was found to be 8.2% [70] (Table 1).

### 3.2. Thyroid Volume and Nodules in SSc

In a recent study conducted by Badak SO et al. in 2022, 86 patients with SSc were examined, 46 of whom had an atrophic thyroid volume, and 40 had a non-decreased thyroid volume. Several statistically significant differences were found between these two groups. Patients with an atrophic thyroid volume were older and had a longer disease duration and higher modified Rodnan skin score (mRSS). Furthermore, a statistically significant correlation was established between the thyroid volumes and TSH levels. Additionally, patients with an atrophic thyroid volume had a higher prevalence of RP, ILD, PAH, and gastrointestinal involvement, all with statistical significance. However, no statistically significant differences were observed between the two groups in terms of sex distribution, the requirement for immunosuppressive therapy, smoking status, or the presence of ANAs and ACAs. Atrophic thyroid volume was more frequently observed in patients with the diffuse type of SSc, while non-decreased thyroid volume was more commonly found in patients with the limited form of SSc. However, no statistical significance was established [71].

Another 2022 study by Meridor K et al. examined a cohort of 50 patients diagnosed with SSc, 10 of whom had a prior diagnosis of thyroid disease. In the SSc patients who did not have thyroid disease, 80% exhibited a standard thyroid size, while only 60% of those with prior thyroid disease had a standard thyroid size. Furthermore, 10% of the SSc patients with thyroid disease had an enlarged thyroid, compared to 17.5% of those without thyroid disease. Additionally, 30% of patients with a prior thyroid disease had a reduced thyroid size, whereas only 2.5% of those without a thyroid disease exhibited a reduced size. However, none of these findings were statistically significant [65]. A total of 22 patients (44%) had 1–6 thyroid nodules, and 24% of the patients presented a nodule >1 cm. Among the study participants, six patients underwent ultrasound-guided fine-needle aspirations, resulting in the diagnosis of papillary thyroid cancer in one patient who had autoimmune hypothyroidism without thyroid autoantibodies. It was observed that a higher proportion of SSc patients without thyroid disease (47%) exhibited thyroid nodules, compared to those with a previous diagnosis of thyroid autoimmune disease (30%). However, this difference did not reach statistical significance. Furthermore, the presence of nodules larger than 1 cm did not differ significantly between patients without thyroid disease and those with known thyroid disease, suggesting that thyroid nodules may be present in SSc patients irrespective of the presence of thyroid disease [65]. 

Antonelli A et al, in a study conducted in 2016 on 327 systemic sclerosis patients, 654 subjects from an iodine-deficient area, and 654 subjects from an iodine-sufficient area, demonstrated that thyroid nodules were more prevalent in control subjects from the iodine-deficient areas and in SSc patients than in controls from iodine-sufficient areas [58].

In a study by Toki, S. et al. in 2014, 210 patients with SSc were included, of whom 30 also presented with AITDs. The study found no statistically significant difference in terms of thyroid volume >20 mL between SSc patients with or without AITDs. However, a higher proportion of patients with SSc and AITDs had a thyroid volume of less than 6 mL compared to those without AITDs, which was statistically significant. The results obtained from this study imply a potential connection between the atrophy of the thyroid gland and the presence of AITDs in SSc patients [59]. No statistically significant difference was established between SSc patients with or without AITDs regarding the presence of thyroid nodules and thyroid cancer [59].

In a longitudinal study conducted by Antonelli, A. et al., encompassing 358 female participants, consisting of 179 individuals with systemic sclerosis (SSc) and 179 controls, the monitoring duration was 73 and 94 months, respectively. Antonelli A and colleagues revealed a statistically significant difference in thyroid volume at the initial assessment, with a lower volume observed in SSc patients than in controls. Although there were slightly more control patients with a thyroid volume exceeding 20 mL, the difference did not reach statistical significance. However, a higher proportion of SSc patients had a thyroid volume of less than 6 mL, which was statistically significant. The findings from the final evaluation were consistent with the initial presentation. A higher proportion of SSc patients continued to exhibit a lower thyroid volume compared to control patients, and this difference was statistically significant. Conversely, more controls had a thyroid volume exceeding 20 mL compared to SSc patients, but no statistical significance was observed. Additionally, more SSc patients had a thyroid volume of less than 6 mL compared to controls, and this difference was found to be statistically significant [62]. The study also assessed the presence of thyroid nodules at the initial and final presentations. The study found that, at the initial presentation, thyroid nodules were identified in 43% of the controls and 39% of patients with SSc, with no statistical significance reached. The results remained consistent at the final presentation, with 50 controls and 44 patients with SSc exhibiting thyroid nodules, again without any statistically significant difference between the two groups. Moreover, SSc patients displayed a more hypoechoic pattern on ultrasound in comparison to controls, and this difference was statistically significant [62].

In another study conducted by Antonelli, A. et al. in 2007, 606 patients were included, comprising 202 SSc patients and 400 controls. The results were also stratified by sex. Similar to the previous study, female SSc patients exhibited a lower thyroid volume than controls, with a statistically significant difference that was not reached when comparing male SSc to male controls. Among female patients, more controls had a thyroid volume exceeding 20 mL compared to control patients, but this difference did not reach statistical significance. Notably, a higher proportion of female SSc patients had a thyroid volume of less than 6 mL compared to female controls, which was statistically significant [64]. In this study, female patients with SSc demonstrated a lower prevalence of thyroid nodules than female control patients. However, this difference did not reach statistical significance [64]. Similarly, a larger percentage of male individuals in the control group showed a higher occurrence of thyroid nodules compared to those with SSc, though this difference lacked statistical significance. Additionally, in male participants, no significant variations were observed between SSc patients and controls concerning thyroid volumes (>20 mL and <6 mL) [64] (Table 2).

### 3.3. Clinical Manifestation in SSc Patients with Thyroid Disorders

In a 2021 study conducted by Paolee Y et al., a total of 200 patients with SSc were examined. Of these, 166 had normal thyroid hormone levels, while 31 patients had clinical or subclinical hypothyroidism and 3 had clinical or subclinical hyperthyroidism. It was observed that RP and weight loss occurred more frequently in patients with normal thyroid hormone levels compared to those with clinical or subclinical hypothyroidism, with a statistically significant difference. However, anemia was more prevalent in patients with SSc and clinical or subclinical hypothyroidism, with statistically significant differences. No statistically significant differences were found between the two groups in terms of age of onset, disease duration, sex, subtype of SSc, BMI, mRSS, digital ulcers, digital gangrene, telangiectasias, calcinosis cutis, salt and pepper skin, edematous skin, tendon friction rubs, hand deformity, arthritis, muscle weakness, esophageal involvement, stomach involvement, ATAs, ACAs, constipation, pulmonary fibrosis, PAH, reduced forced vital capacity less than 70%, pericardial effusions, and reduced left ventricular ejection fraction less than 50%. These findings suggest that these manifestations appear independently of thyroid disease in SSc patients [72]. 

Another study conducted by Triggianese P et al. included 77 pregnant patients with SSc and 50 age-matched controls. Within the SSc group, 23 patients presented pregnancy failures. Overall, the prevalence of pregnancy failure was similar in SSc patients when compared to controls. However, the prevalence of preterm delivery and intrauterine growth restriction (IUGR) was significantly higher in SSc patients, while infertility and recurrent spontaneous abortion (RSA) were significantly higher in control patients. When considering thyroid disorders, 19 SSc patients presented with HT, 16 with non-toxic multinodular goiter, 5 with hypothyroidism, and 1 with thyroid cancer. The study found that women with thyroid disorders were more likely to have a limited form of SSc compared to those without thyroid disorders. Conversely, female patients without thyroid disorders were more likely to have the diffuse subtype of SSc. Both of these findings were statistically significant. Furthermore, digital ulcers and esophageal reflux were less common in SSc patients with thyroid disease than those without, with a statistically significant difference. The study also revealed that HT, ANAs positivity, and dcSSc were more prevalent in female patients with pregnancy failures compared to those without, while lcSSc, ACAs, and non-toxic multinodular goiter were more frequent in women without pregnancy failures. Interestingly, the study did not find a higher prevalence of thyroid dysfunctions in female patients with SSc compared to the control group, and no disease progression was identified during pregnancy [73]. 

In a separate study conducted by Posselt RT and colleagues, there was no statistically significant difference found among SSc patients with anti-TPO antibodies with hypothyroidism, those with anti-thyroid antibodies without hypothyroidism, and SSc patients without anti-thyroid antibodies when considering the mRRSS or the Medsger index [58].

In a cross-sectional study conducted by Wielosz E et al., 86 patients with SSc were included; 27 patients had anti-TPO and anti-Tg antibodies, while 59 patients had no thyroid autoimmune disease antibodies. No significant differences were found in the prevalence of calcinosis, digital ulcers, gastrointestinal involvement, ILD, primary heart involvement, PAH on echocardiography, kidney involvement, SRC, joint involvement, overlap syndromes, myalgia, myositis, anti-Scl-70 antibodies, anti-Pm/Scl, ARAs, anti Tj1/Th0, anti-Ku, anti-Nor 90 or ACAs between SSc patients with or without anti-thyroid antibodies. The prevalence of anti-Ro52 antibodies was higher in the SSc group with positive anti-TPO antibody titers than in the SSc group without anti-TPO titers [66].

In a separate study conducted by Bagnato et al. involving 105 female patients (70 with SSc and 35 with HT), it was found, with statistical significance, that female patients with HT were younger than those with SSc (both lcSSc and dcSSc). Additionally, SSc patients exhibited more frequent positivity for ANAs, SCL70, and ACAs than HT patients, which reached statistical significance. Furthermore, the mRSS was significantly elevated within the SSc patient group compared to HT patients. Notably, the levels of TSH positively correlated, and the levels of FT3 and FT4 inversely correlated with the mRSS, all demonstrating statistical significance. Additionally, TSH and FT4 showed a direct and inverse correlation, respectively, with the mRSS in both lcSSc and dcSSc, as reported by Bagnato. The study also demonstrated that serum TSH levels were significantly higher in the dcSSc group compared to the lcSSc group [67].

A study by Costa, C.C. et al., involving 56 patients diagnosed with SSc, 11 presenting HT, found no significant difference between patients with and without HT in terms of diffuse or limited types of SSc [74].

A study by Toki, S. et al. in 2014 included 210 patients with SSc, of whom 30 also had AITDs. Among these 30 patients, 1 had GD, and the remaining 29 had HT. The study did not find any statistically significant differences between the two groups in terms of the type of SSc (limited or diffuse), the presence of anti-U1 RNP, anti-SS-A, anti-SS-B antibodies, PAH, digital ulcerations, and cardiovascular involvement. Although ILD was less prevalent in SSc patients with AITDs, the difference did not reach statistical significance. It was observed that patients with SSc and autoimmune thyroiditis had a higher frequency of positive ANAs and ACAs compared to patients without AITDs. Interestingly, the study revealed that ATAs’ characteristics of SSc were found to be positive more frequently in patients without autoimmune thyroiditis, and this difference was statistically significant. Furthermore, among SSc patients, those with clinical AITDs exhibited a significantly higher modified mRSS for the face compared to SSc patients with subclinical AITDs or without AITDs. Sjogren Syndrome was found to be more prevalent in patients with SSc and autoimmune thyroiditis, with the difference being statistically significant [59].

The investigation conducted by Avouac J et al., involving 1132 patients diagnosed with SSc, of whom 70 also presented autoimmune thyroiditis, found no significant differences in terms of age, sex, disease duration, limited cutaneous subtype, digital ulcers, PAH, ANAs, ATAs, ACAs positivity, decreased diffusing capacity for carbon monoxide/alveolar volume (DLCO/AV), immunosuppressive therapy, and decreased forced vital capacity among the SSc patients with or without AITDs. SSc patients without AITDs were more likely to present pulmonary fibrosis on computer tomography scans, the differences being statistically significant [68].

Ugurlu S and colleagues demonstrated a significant correlation between anti-TPO and anti-Tg antibodies with anti-Scl-70 antibodies (*p* = 0.003; *p* < 0.001) [75] (Table 3).

### 3.4. SSc, Thyroid Autoimmune Disorders, and Familial Autoimmunity

Research has shown that individuals with a family history of autoimmune diseases are more likely to develop autoimmune diseases themselves. The results of a study conducted in Taiwan highlighted that individuals who were relatives of patients with SSc faced an increased likelihood of developing SSc themselves [76]. Moreover, the relatives of the SSc patients were at higher risk of developing other autoimmune diseases besides SSc, such as rheumatoid arthritis, AITDs, Sjogren syndrome, and primary biliary cirrhosis [77].

A recent prospective study by Meridor K et al. in 2022 investigated the prevalence of thyroid autoimmune disease in first-degree relatives of patients with SSc. The study found that 40% of the first-degree relatives of patients previously diagnosed with thyroid disease had thyroid autoimmune disease, compared to 31% of patients with SSc without thyroid disease. However, the difference between the two groups did not reach statistical significance [65].

In addition, research conducted by Antonelli A et al. in 2016 revealed that familial thyroid disease was more prevalent in the control patients from iodine-deficient areas (46%) compared to both SSc patients (38%) and control patients from iodine-sufficient regions (18%). This difference was found to be statistically significant [58].

Furthermore, in a 2012 study by Koumakis E et al., the number of families with at least one autoimmune disease was higher in the families of the SSc patients (32.4%) compared to 19.6% in the control families. The difference proved statistically significant. AITDs were identified in 13.1 families of SSc patients. More first-degree relatives of the SSc patients (40%) developed AITDs compared to the first-degree relatives of the controls, with a statistically significant difference. HT was more frequent in the first-degree relatives of patients compared to first-degree relatives of controls, 26% versus 3%, the difference reaching statistical significance. GD was also more frequent in the first-degree relatives of the SSc patients. However, the difference was not statistically significant [78] (Table 4).

## 4. Discussion

Several investigations have proposed a higher incidence of thyroid dysfunctions in patients with SSc, yielding conflicting outcomes.

Regarding the prevalence of clinical hypothyroidism in SSc patients, the numbers varied from 4% to 33.3% [63,64]; several studies have demonstrated that clinical hypothyroidism occurs with a higher prevalence in SSc patients when compared to controls [57,58,62,64]. Other investigations did not find a statistically significant higher prevalence of clinical hypothyroidism in SSc patients when compared to controls; however, they demonstrated that patients with SSc exhibited higher levels of TSH, subclinical hypothyroidism, and a greater prevalence of thyroid dysfunction [62]. Moreover, clinical and subclinical hypothyroidism were more prevalent in patients with SSc when compared to controls residing in both iodine-sufficient and -deficient areas [58]. Furthermore, the FT4 levels of SSc patients were found to be significantly lower than those of controls [63].

When patients with systemic sclerosis with previously diagnosed thyroid disease were compared to SSc patients without thyroid disease, both clinical and subclinical hypothyroidism were more frequent in patients with SSc with AITDs [59].

Several studies could not reach a consensus about an association between systemic sclerosis and hyperthyroidism [62,64]. GD was more prevalent in female SSc patients compared to controls in one study [64]; nevertheless, other studies could not establish an association. In contrast to prior research, a recent investigation demonstrated that not only is hyperthyroidism statistically significantly higher in SSc patients but hyperthyroidism is aldo associated with a higher rate of all-cause mortality when compared to controls with and without hyperthyroidism and SSc patients without hyperthyroidism [56].

Various studies have explored the prevalence of autoimmune thyroid disorders (HT and GD) in SSc patients, yielding different results, the prevalence varying from 7.6% [70] to 14.4% [69]. It was observed that anti-TPO antibodies, but not anti-Tg antibodies, associated with hypothyroidism were found to be more frequent in SSc patients compared to controls [57,62]. This trend aligns with the existing literature, including the study by Marasini B and colleagues. Their research specifically demonstrated a greater risk of developing hypothyroidism in female SSc patients with the presence of anti-TPO antibodies [79]. Other studies revealed that both anti-TPO and anti-Tg antibodies are found with a higher prevalence in systemic sclerosis patients [58]. Earlier research, such as a study conducted by Molteni M and colleagues in 1997, supports these findings by demonstrating that 19% and 12% of SSc patients displayed anti-TPO antibodies and anti-Tg antibodies, respectively. In comparison to the control group, patients with scleroderma have a significantly higher likelihood of displaying anti-TPO antibodies with hypothyroidism (3.4-fold) and anti-TPO or/and anti-Tg without hypothyroidism (3.1-fold) [57]. Comparable outcomes were observed for patients with systemic lupus erythematosus but not for those with rheumatoid arthritis or ankylosing spondylitis [57]. Female SSc patients have significantly higher levels of anti-TPO antibodies compared to controls and a higher percentage of patients with positive anti-TPO antibodies [64]. The same trend was observed in male patients, where anti-TPO antibody levels and the percentage of patients presenting with anti-TPO antibodies and thyroid autoimmunity are significantly higher in male SSc patients compared to male controls [64].

In individuals with SSc, the occurrence of thyroid autoantibodies was more frequent among those concurrently affected by AITDs in comparison to those without thyroid-related conditions [59]. As expected, when comparing the prevalence of thyroid autoimmune antibodies in SSc patients to patients suffering from HT, the prevalence was statistically higher in the patients suffering from HT [67].

Regarding the association of thyroid cancer and SSc, one study from 2016 demonstrated a higher prevalence of papillary thyroid cancer in SSc patients when compared to controls. These patients had a higher anti-TPO antibody prevalence, with statistical significance of particular importance. The association was not identified in other studies cited herein [58,59,60,65].

Thyroid volume was significantly lower in SSc patients compared to controls in some studies [62,64]. A higher proportion of SSc patients had a thyroid volume of less than 6 mL [62], and a higher proportion of female SSc patients had a thyroid volume of less than 6 mL compared to female controls [64]. The same applied to patients with SSc and autoimmune thyroid disease who presented more frequently with thyroid volumes less than 6 ml [59]. Other studies demonstrated that control patients have thyroid volumes of more than 20 mL when compared to SSc patients; however, no statistical significance was established [59,62,64].

Furthermore, one study postulated that atrophic thyroid volume is more frequently observed in patients with dcSSc, while non-decreased thyroid volume is more commonly found in patients with lcSSc. However, this conclusion did not reach statistical significance [71]. No statistically significant differences were observed between SSc patients with atrophic and non-atrophic thyroid volumes in terms of sex distribution, the requirement for immunosuppressive therapy, smoking status, or the presence of ANAs and ACAs [71].

Regarding the presence of thyroid nodules in SSc patients, several studies demonstrated that the prevalence of thyroid nodules is higher in control patients than in SSc patients. Even though the difference was not significant, these results suggest that thyroid nodules appear more frequently in the general population than in patients with SSc [62,64]. When patients with SSc and AITDs were compared to SSc patients without autoimmune disease, more patients without thyroid disease presented thyroid nodules [65], with no statistical significance. These results suggest that thyroid nodules may be present in SSc patients irrespective of the presence of AITDs. The same outcome was observed when comparing the incidence of thyroid nodules in SSc patients with or without AITDs, where more SSc patients without AITDs presented thyroid nodules [59].

The majority of the included studies did not identify significant associations between thyroid dysfunctions, autoimmunity, and the features of SSc. These findings suggest that, in the context of SSc, there may be limited or no observed connections between thyroid-related issues and the distinctive features of the disease.

A study conducted on pregnant patients with systemic sclerosis, a few of whom also presenting autoimmune thyroiditis, found that women with thyroid disorders were more likely to have the limited form of SSc compared to those without thyroid disorders. Conversely, female patients without thyroid disorders were more likely to have the diffuse subtype of SSc [73]. However, other studies found no significant difference between patients with and without HT in terms of diffuse or limited types of SSc [59,68,72,74]. The same study conducted on pregnant SSc patients demonstrated that digital ulcers and esophageal reflux were more common in patients without thyroid disease compared to those with autoimmune thyroiditis, with a statistically significant difference [73]. Furthermore, HT, ANAs positivity, and dcSSc were more prevalent in female SSc patients with pregnancy failures, while lcSSc, ACAs, and non-toxic multinodular goiter were more frequent in women without pregnancy failures [73].

Another study found no significant differences in terms of age, disease duration, limited cutaneous subtype, PAH, ANAs, ATAs, ACAs positivity, digital ulcers, immunosuppressive therapy, decreased DLCO/AV, and decreased forced vital capacity among SSc patients with or without autoimmune thyroiditis [68]. However, patients without AITDs were more likely to present pulmonary fibrosis on CT scans [68]. An additional investigation demonstrated no differences in the prevalence of age of onset, disease duration, sex, the subtype of SSc, BMI, mRSS, digital ulcers, digital gangrene, telangiectasias, calcinosis cutis, salt and pepper skin, edematous skin, tendon friction rubs, hand deformity, arthritis, muscle weakness, esophageal involvement, stomach involvement, constipation, pulmonary fibrosis, PAH, reduced forced vital capacity less than 70%, pericardial effusions, and reduced left ventricular ejection fraction less than 50% between SSc patients with normal thyroid hormone levels and SSc patients with clinical or subclinical hypothyroidism. These findings suggest that these manifestations appear independently of thyroid disease in SSc patients [72]. Nevertheless, there was one study in which the mRSS correlated directly with TSH levels and indirectly with FT4 levels [67], suggesting that the degree of skin sclerosis is higher in patients with high TSH and low FT4 levels. Another study revealed no differences in mRSS among SSc patients with clinical AITDs, subclinical AITDs, and those without AITDs. However, when assessing facial skin sclerosis, the skin score was significantly higher in SSc patients with clinical AITDs compared to those with subclinical AITDs and those without AITDs [59].

Counter to expectations, RP and weight loss were found to manifest more frequently in SSc patients with normal thyroid hormone levels when compared to SSc patients with clinical or subclinical hypothyroidism; however, anemia was more frequent in SSc patients with clinical or subclinical hypothyroidism, with a statistical significance [72].

Two additional studies illustrated no significant differences in the prevalence of calcinosis, digital ulcers, gastrointestinal involvement, ILD, primary heart involvement, PAH on echocardiography, kidney involvement, SRC, joint involvement, overlap syndromes, myalgia, myositis, anti-Scl-70, ACAs, ARAs, anti Th1/Th0, anti-Pm/Scl, anti-Ku, and anti-NOR 90 antibodies in SSc patients with anti-thyroid antibodies when compared to SSc patients with no anti-thyroid antibodies [66] and no significant difference in the presence of anti-RNP, anti-SS-A, anti-SS-B antibodies, anti-U1 RNP, PAH, digital ulcerations, and cardiovascular involvement between patients with and without HT [59]. More patients with SSc and autoimmune thyroiditis had a higher frequency of positive ANAs and ACAs compared to patients without AITDs [59]. Additionally, another study found out that patients with SSc and AITDs had a higher frequency of ACAs positivity compared to patients without AITDs, which was statistically significant [68]. However, ATAs were found to be more frequently positive in patients without autoimmune thyroiditis, and this difference was statistically significant [59].

Sjogren Syndrome was found to occur more frequently in patients with SSc and autoimmune thyroiditis, with a statistically significant difference [59].

On the subject of thyroid volume and clinical manifestations of SSc, it was noted that patients with an atrophic thyroid volume are older and have a longer duration of disease, higher mRSS, and significantly lower levels of TSH compared to SSc patients with a non-decreased thyroid volume and a higher prevalence of RP, ILD, PAH, and gastrointestinal involvement, all with statistical significance [71].

Concerning the topic of the risk of thyroid disorders in first-degree relatives of SSc patients, one study demonstrated a higher risk for developing HT [78]; however, the results did not attain statistical significance in other investigations [58,65].

## 5. Conclusions

The association between SSc and thyroid disorders is complex and multifaceted.

Thyroid dysfunction can influence the course of systemic sclerosis and vice versa. Managing patients with both systemic sclerosis and thyroid disorders can present challenges and recognizing the association prompts healthcare providers to implement targeted screening for thyroid disorders in patients with systemic sclerosis. Regular monitoring is essential to detect and address thyroid abnormalities, minimizing potential complications promptly.

Both SSc and thyroid disorders are autoimmune conditions, and further investigations may provide insights into common autoimmune mechanisms and pathways. This knowledge contributes to a broader understanding of autoimmune diseases in general.

Unraveling the link between SSc and thyroid disorders provides a basis for in-depth research into the shared pathogenic mechanisms. This research can lead to targeted therapies addressing the underlying causes of both conditions.

Moreover, identifying patients with both systemic sclerosis and thyroid disorders may serve as a prognostic indicator. Understanding the impact of concurrent conditions on disease outcomes allows for tailored treatment plans and a better prediction of long-term prognosis.

Exploring the association between systemic sclerosis and thyroid disorders goes beyond clinical management. It delves into the intricate interplay of autoimmune processes, guides screening and monitoring practices, and opens avenues for targeted research to improve diagnosis and treatment strategies for affected individuals.

## Figures and Tables

**Table 1:** Thyroid dysfunction, autoimmunity, and cancer in SSc patients.

Study, Year, Reference	Design	Patients	Study Findings
Zhou Z 2022 [61]	Retrospective study	N = 8120 P with autoimmune diseases N1 = 375 P with SSc	Thyroid cancer: N SIR = 6.45 (95% CI = 4.81–8.83); N1 = not established
Meridor K 2022 [65]	Prospective study	N = 50 P with SSc N1 = 10 (20%) P with thyroid disease previously diagnosed N2 = 40 (80%) P not previously diagnosed with thyroid disease N3 = 40 (80%) P with diffuse SSc N4 = 10 (20%) P with limited SSc	Exposed to neck radiation: N = 0 Hemi-thyroidectomy: N = 2 P Papillary thyroid cancer in N1 = 1 P Thyroid autoimmune disease in first-degree relatives N = 16 P (33%); N1 = 4 P (40%); N2 = 12 P (31%); [*p*-value = 0.420] anti-TPO antibodies: N = 8 P (17%); N1 = 3 P (30%); N2 = 5 P (13%); [*p*-value = 0.207] anti-TG antibodies: N = 5 P (10%); N1 = 3 P (30%); N2 = 2 P (5%); [*p*-value = 0.054] TSH (nl 0.23–4 mIU/L), median (IQR): N1 = 2.3 (1.7–3.9); N2 = 1.8 (1.3–2.8); [*p*-value = 0.163] FT4 (nl 0.8–2 ng/dL), median (IQR): N1 = 1.0 (0.9–1.1); N2 = 1.0 (0.9–1.1); [*p*-value = 0.381]
Akuka A 2019 [56]	Cross-sectional study	N = 15141 P N1 = 2431 P with SSc N2 = 12,710 P controls N3 = 12,452 P controls without hyperthyroidism N4 = 258 P controls with hyperthyroidism N5 = 2357 P with SSc without hyperthyroidism N6 = 74 P with SSc and hyperthyroidism	Hyperthyroidism (*n*; %): N1 = 74 (3%); N2 = 258 (2%); [*p*-value < 0.0001]. All-cause mortality (n; %): N3 = 1532 (12.3%); N4 = 57 (22.1%); N5 = 609 (25.8%); N6 = 28 (37.8%); [*p*-value < 0.0001] Age (mean ± SD; median): N3 = 63.27 ± 18.12; 66; N4 = 71.83 ± 13.96; 75; N5 = 62.49 ± 17.98; 66; N6 = 68.92 ± 14.14; 71; [*p*-value < 0.0001] Age at diagnosis (mean ± SD; median): N3 = 54.37 ± 18.66; 57; N4 = 62.37 ± 14.84; 64; N5 = 54.57 ± 18.76; 57; N6 = 61.04 ± 14.26; 62.5; [*p*-value < 0.0010] Gender (female; %): N3 = 10161 (81.6%); N4 = 229 (88.8%); N5 = 1919 (81.4%); N6 = 68 (91.9%); [*p*-value = 0.0030]
Posselt RT 2017 [57]	Cross-sectional study	N = 790 P N1 = 649 P with autoimmune disease N2 = 58 P with SSc N3 = 141 P controls	Female gender: N2 = 55 (94.8%); N3 = 130 (92.1%); Hypothyroidism: N2 = 16 (27.5%); N3 = 16 (11.3%); At least one anti-thyroid antibody: N2 = 12 (15%); N3 = 23 (16.3%). Anti-TPO with hypothyroidism: N2 = 12 (20.6%); N3 = 8 (5.6%). Anti-TPO without hypothyroidism: N2 = 3 (5.1%); N3 = 12 (8.5%). Anti-Tg without hypothyroidism: N2 = 3 (5.1%); N3 = 7 (4.9%). Anti-TPO with hypothyroidism: N2: OR = 3.4 (1.06–10.80); *p* = 0.03. Anti-TPO/anti-Tg without hypothyroidism: N2: OR = 3.1 (1.11–0.13); *p* = 0.03.
Wielosz E 2016 [66]	Cross-sectional study	N = 86 P with SSc N1 = 32 P with diffuse SSc N2 = 54 P with limited SSc N3 = 27 P with SSc and anti-TPO/anti-Tg-positive N4 = 59 P with SSc and anti-TPO/anti-Tg-negative	Anti-TPO: N = 22 P (26%); N1 = 8 P (25%); N2 = 14 P (26%); [*p*-value = NS] Anti-Tg: N = 22 P (26%); N1 = 9 P (28%); N2 = 13 P (24%); [*p*-value = NS] Anti-TPO or Anti-Tg: N = 27 P (31%); N1 = 11 P (34%); N2 = 16 P (30%); [*p*-value = NS] Anti-TPO and Anti-Tg: N = 17 P (20%); N1 = 6 P (19%); N2 = 11 P (20%); [*p*-value = NS] AITDs: N = 26 P (30%); N1 = 10 P (31%); N2 = 16 P (30%); [*p*-value = NS] Anti-Scl-70 antibodies: N3 = 11 P (41%); N4 = 18 P (33%); [*p*-value = NS] ACAs: N3 = 6 P (22%); N4 = 13 P (16%); [*p*-value = NS]
Antonelli A 2016 [58]	Cross-sectional study	N = 1635 P N1 = 327 P with SSc N2 = 654 P in the control group from an iodine-deficient area N3 = 654 P from an iodine-sufficient area N4 = 321 P with SSc without papillary thyroid cancer N5 = 6 P with SSc and papillary thyroid cancer	Age, mean (s.d.) years: N1 = 54 (14); N2 = 55 (11); N3 = 54 (13); [*p*-value = 0.724] Men/women %: N1 = 8/92; N2 = 8/92; N3 = 8/92; [*p*-value = 1] TSH, median (range), mlU/l: N1 = 3.2 (0.01–51.4); N2 = 1.2 (0.01–8.7); N3 = 1.4 (0.1–9.6); [*p*-value = 0.0013] FT4, mean (s.d.), pmol/L: N1 = 9.5 (5.1); N2 = 11.4 (3.1); N3 = 12.2 (2.9). [*p*-value = 0.537] FT3, mean (s.d.), pmol/L: N1 = 4.6 (1.6); N2 = 5.1 (1.5); N3 = 4.9 (2.1). [*p*-value = 0.421] Hypothyroidism, TSH > 4 mlU/L, %: N1 = 20; N2 = 3; N3 = 5. [*p*-value < 0.001] Hyperthyroidism, TSH < 0.3 mlU/L, %. N1 = 4; N2 = 3; N3 = 2. [*p*-value = 0.172] Iodine deficiency, %: N1 = 57; N2 = 87%; N3 = 19%. [*p*-value < 0.0001] Familial thyroid disease, %: N1 = 38; N2 = 46; N3 = 18. [*p*-value < 0.0001] Anti-Tg, median (range), kIU/L: N1 = 135 (2–1213); N2 = 31 (3–321); N3 = 47 (1–476). [*p*-value = 0.041] Anti-TPO, median (range), kIU/L: N1 = 115 (9–2132); N2 = 29 (1–423); N3 = 28 (4–432). [*p*-value = 0.0005] Anti-Tg, %: N1 = 18; N2 = 8; N3 = 12. [*p*-value = 0.0001] Anti-TPO, %: N1 = 35; N2 = 7; N3 = 9. [*p*-value = 0.0001] PTC, n: N1 = 6; N2 = 1; N3 = 1. [*p*-value = 0.007] Anti-Tg (±), n: N4 = 59/262; N5 = 3/3; [*p*-value = 0.084] Anti-TPO (±), n: N4 = 109/212; N5 = 5/1; [*p*-value = 0.021]
Bagnato 2015 [67]	Cross-sectional study	N = 105 P N1 = 70 P with SSc N2 = 35 P controls with HT N3 = 30 P with dcSSc N4 = 40 P with lcSSc	Anti-TPO-positive n (%): N1 = 8 (11); N2 = 22 (62); N3 = 2 (7); N4 = 6 (15); [*p*-value < 0.0001] Anti-Tg-positive n (%): N1 = 17 (24); N2 = 18 (51); N3 = 8 (26); N4 = 9 (22); [*p*-value < 0.0001] FT3 (pg/mL): N1 = 2.91 ± 0.65; N2 = 2.79 ± 0.81; N3 = 2.74 ± 0.43; N4 = 3.04 ± 0.76; [*p*-value = NS] FT4 (pmol/L): N1 = 14.39 ± 3.94: N2 = 12.19 ± 2.28; N3 = 13.87 ± 3.13; N4 = 14.78 ± 4.45; [*p*-value = 0.0037] TSH (mIU/L): N1 = 2.07 ± 1.10; N2 = 5.41 ± 9.34; N3 = 2.7 ± 1.06; N4 = 1.6 ± 0.88; [*p*-value = 0.0038]
Toki S 2014 [59]	Observational retrospective study	N = 210 P with SSc N1 = 30 P with SSc and AITDs N2 = 180 P with SSc without AITDs	GD (%): N = 0.48 (1/210); N1 = 3.3 (1/30); HT (%): N = 13.8 (29/210); N1 = 96.7 (29/30). Sex: Male (%): N = 11.0 (23/210); N1 = 0 (0/30); N2 = 12.8 (23/180); Female (%): N = 89.0 (187/210); N1 = 100 (30/30); N2 = 87.2 (157/180); [*p*-value = 0.038] Clinical hypothyroidism (%): N = 9.5 (20/210); N1 = 66.7 (20/30). Subclinical hypothyroidism (%): N = 4.3 (9/210); N1 = 30.0 (9/30). Anti-Tg (%): N = 19.5 (41/210); N1 = 66.7 (20/30); N2 = 14.3 (21/180). Anti-TPO (%): N = 22.4 (47/210); N1 = 73.3 (22/30); N2 = 14.8 (25/180). Anti-Tg or anti-TPO (%): N = 28.6 (60/210); N1 = 100 (30/30); N2 = 16.7 (30/180); Ultrasonography findings: Thyroid cancer (%): N = 1.7 (1/63); N1 = 0 (0/30); N1 = 3 (1/33); [*p*-value = 0.345] Type: lcSSc (%): N = 70.5 (148/210); N1 = 80.0 (24/30); N2 = 68.9 (124/180). dcSSc (%): N = 29.5 (62/210): N1 = 20.0 (6/30); N2 = 31.1 (56/180) [*p*-value = 0.217]
Antonelli A 2013 [62]	Longitudinal study	N = 358 P N1 = 179 P with SSc N2 = 179 P without SSc in control group	Initial thyroid status: Age, y (SD): N1 = 50 (13); N2 = 52 (10); [*p*-value = NS] Sex (female): N1 = 179; N2 = 179; [*p*-value = NS] TSH, microU/mL (SD): N1 = 2.1 (0.7); N2 = 1.1 (0.6); [*p*-value = 0.043] FT4, pmol/L (SD): N1 = 115 (4.1); N2 = 122 (3.1); [*p*-value = NS] FT3, pmol/L (SD): N1 = 4.7 (1.9); N2 = 5.0 (1.1); [*p*-value = NS] Subclinical hypothyroidism, %: N1 = 0; N2 = 0 [*p*-value = NS] Clinical hypothyroidism, %: N1 = 0; N2 = 0 [*p*-value = NS] Subclinical hyperthyroidism, %: N1 = 0; N2 = 0 [*p*-value = NS] Thyroid dysfunctions, %: N1 = 0; N2 = 0 [*p*-value = NS] Anti-Tg, IU/mL (SD): N1 = 72 (134); N2 = 27 (85%); [*p*-value = NS] Anti-TPO, IU/mL (SD): N1 = 91 (169); N2 = 15 (35); [*p*-value = 0.026] GD, %: N1 = 0; N2 = 0 [*p*-value = NS] Anti-Tg, %: N1 = 11; N2 = 7; [*p*-value = NS] Anti-TPO, %: N1 = 21; N2 = 12; [*p*-value = 0.022] Thyroid autoimmunity, %: N1 = 24; N2 = 12; [*p*-value = 0.002] Thyroid cancer, %: N1 = 0; N2 = 0; [*p*-value = NS] Last evaluation: Age, y (SD): N1 = 56 (13); N2 = 60 (10); [*p*-value = NS] Sex (female): N1 = 179; N2 = 179; [*p*-value = NS] TSH, microU/mL (SD): N1 = 2.9 (4.1); N2 = 1.3 (1.1); [*p*-value = 0.018] FT4, pmol/L (SD): N1 = 110 (4.6); N2 = 121 (3.4); [*p*-value = NS] FT3, pmol/L (SD): N1 = 4.5 (1.9); N2 = 4.9 (1.2); [*p*-value = NS] Subclinical hypothyroidism, %: N1 = 7.8; N2 = 1.7 [*p*-value = 0.006] Clinical hypothyroidism, %: N1 = 1.7; N2 = 0.56 [*p*-value = NS] Subclinical hyperthyroidism, %: N1 = 2.2; N2 = 1.7 [*p*-value = NS] Anti-Tg, IU/mL (SD): N1 = 94 (215); N2 = 36 (96%); [*p*-value = NS] Anti-TPO, IU/mL (SD): N1 = 125 (256); N2 = 23 (52); [*p*-value = 0.013] GD, %: N1 = 1.1; N2 = 0 [*p*-value = NS] Thyroid dysfunctions, %: N1 = 13; N2 = 4 [*p*-value = 0.001] Anti-Tg, %: N1 = 13; N2 = 9; [*p*-value = NS] Anti-TPO, %: N1 = 27; N2 = 13; [*p*-value = 0.002] Thyroid autoimmunity, %: N1 = 34; N2 = 16; [*p*-value < 0.001] Thyroid cancer, %: N1 = 1.1; N2 = 0; [*p*-value = NS] New cases: Subclinical hypothyroidism: N1 = 14 (12.8); N2 = 3 (2.1); [*p*-value = 0.006] Clinical hypothyroidism: N1 = 3 (2.7); N2 = 1 (0.7); [*p*-value = NS] Hypothyroidism: N1 = 17 (15.5); N2 = 4 (2.8); [*p*-value = 0.003] Subclinical hyperthyroidism: N1 = 4 (3.6); N2 = 3 (2.1); [*p*-value = NS] Hyperthyroidism: N1 = 6 (5.4); N2 = 3 (2.1); [*p*-value = NS] Thyroid dysfunction: N1 = 23 (21); N2 = 7 (4.1); [*p*-value = 0.001] GD: N1 = 2 (1.8); N2 = 0 (0); [*p*-value = NS] Thyroid dysfunction: N1 = 23 (21); N2 = 7 (4.1); [*p*-value = 0.001] Anti-Tg: N1 = 4 (3.6); N2 = 3 (2.1); [*p*-value = NS] Anti-TPO: N1 = 11 (11); N2 = 3 (2.8); [*p*-value = 0.04]
Avouac J 2010 [68]	Observational study	N = 1132 P with SSc N1 = 585 P with SSc from France N2 = 547 P with SSc from Italy N3 = 70 P with SSc and autoimmune thyroiditis N4 = 1062 P with SSc without autoimmune thyroiditis	Autoimmune thyroiditis [*n* (%)]: N = 70 (6); N1 = 23 (4); N2 = 47 (8.5); [*p*-value = 0.025] Female, n (%): N3 = 68/70 (97%); N4 = 921/1062 (87%); [*p*-value = 0.04] Age, mean ± SD, yrs: N3 = 62 ± 13; N4 = 63 ± 13; [*p*-value = 0.9] Disease duration, yrs: N3 = 11 ± 9; N4 = 12 ± 9; [*p*-value = 0.5]
Caramaschi P 2007 [69]	Cross-sectional study	N = 118 P with SSc	Concomitant autoimmune disease (%): N = 38 (32.2%). Autoimmune thyroiditis total (%): N = 17 (14.4%). Autoimmune thyroiditis before SSc diagnosis: N = 7. Autoimmune thyroiditis at diagnosis of SSc: N = 2. Autoimmune after SSc diagnosis: N = 8.
Antonelli A 2007 [64]	Cross-sectional study	N = 606 P N1 = 202 P with SSc N2 = 404 P controls N3 = 184 P females with SSc N4 = 18 P males with SSc N5 = 368 P female control patients N6 = 36 P male control patients	Female patients: Age (years): N3 = 55 ± 12; N5 = 54 ± 9; [*p*-value = NS]. TSH (mU/mL): N3 = 3.6 ± 11.6; N5 = 1.6 ± 1.4; [*p*-value = 0.0013]. FT4 (pmol/L): N3 = 115 ± 5.1; N5 = 117 ± 26; [*p*-value = NS]. FT3 (pmol/L): N3 = 4.6 ± 1.5; N5 = 4.8 ± 2; [*p*-value = NS]. Subclinical hypothyroidism: N3 = 31/153 (17%); N5 = 22/346 (6%); [*p*-value = 0.0001]. Clinical hypothyroidism: N3 = 7/177 (4%); N5 = 1/367 (0.3%); [*p*-value = 0.004]. Subclinical hyperthyroidism: N3 = 6/178 (3.3%); N5 = 22/346 (6%); [*p*-value = NS]. Anti-Tg (IU/mL): N3 = 121 ± 207; N5 = 66 ± 206; [*p*-value = NS]. Anti-TPO (IU/mL): N3 = 115 ± 491; N5 = 24 ± 62; [*p*-value = 0.0005]. GD: N3 = 3 (1.6%); N5 = 0; [*p*-value = 0.0140]. Anti-Tg: N3 = 35/149 (19%); N5 = 59/309 (16%); [*p*-value = NS]. Anti-TPO: N3 = 72/112 (39%); N5 = 70/298 (19%); [*p*-value = 0.0001]. Thyroid autoimmunity: N3 = 107/77 (58%); N5 = 100/268 (27%); [*p*-value = 0.0001]; Male patients: Age (years): N4 = 51 ± 18; N6 = 50 ± 11; [*p*-value = NS]. TSH (mU/mL): N4 = 1.8 ± 0.9; N6 = 1.4 ± 0.8; [*p*-value = 0.0013]. FT4 (pmol/L): N4 = 118 ± 24; N6 = 124 ± 24; [*p*-value = NS]. FT3 (pmol/L): N4 = 4.4 ± 1; N6 = 4.9 ± 0.6; [*p*-value = NS]. Subclinical hypothyroidism: N4 = 1/17 (6%); N6 = 0; [*p*-value = NS]. Subclinical hyperthyroidism: N4 = 0; N6 = 1/35 (3%); [*p*-value = NS]. N4 = 3/15 (17%); N6 = 8/28 (22%); [*p*-value = NS]. Anti-Tg (IU/mL): N4 = 43 ± 111; N6 = 14 ± 13; [*p*-value = NS]. Anti-TPO (IU/mL): N4 = 70 ± 80; N5 = 30 ± 20; [*p*-value = 0.0062]. Anti-Tg: N4 = 3/15 (17%); N6 = 2/34 (6%); [*p*-value = NS]. Anti-TPO: N3 = 3/15 (17%); N6 = 0; [*p*-value = 0.0117]. Thyroid autoimmunity: N4 = 7/11 (39%); N6 = 2/34 (6%); [*p*-value = 0.0019];
Biro E 2006 [70]	Retrospective study	N = 1517 P with autoimmune diseases N1 = 119 P with SSc	HT: N = 86 (5.7%); N1 = 5 (4.2%) GD: N = 39 (2.6%); N1 = 4 (3.4%) All AITDs: N = 125 (8.2%); N1 = 9 (7.6%) EG: N = 44 (2.9%); N1 = 5 (4.2%) All thyroid (HT + GD + EG): N0 = 169 (11.1%); N1 = 14 (11.8%) HT relative prevalence: N1 = 0.042 (4.2%). GD relative prevalence: N1 = 0.033 (3.3%).
Derk CT 2006 [60]	Observational study	N = 769 P with SSc	Thyroid cancer: N = 2 4.34 (–1.66–10.34) The number was too small to reach statistical significance
Shahin AA 2002 [63]	Cross-sectional study	N = 38 P N1 = 24 P with SSc N2 = 23 P females with SSc N3 = 15 P female control patients	Hypothyroidism: N1 = 8/24 (33.3%). FT4: N1 = 7.46 ± 2.7; N3 = 10.5 ± 1.8; [*p*-value < 0.001]. FT4 Hyperthyroidism: N = 0. FT3: N1 = 4.8 ± 2.3; N3 = 5.3 ± 1.2; [*p*-value = NS] T3 thyrotoxicosis n (%): N1 = 3 (12.5%)

Abbreviations: P, patients; SSc, systemic sclerosis; lcSSc, limited cutaneous systemic sclerosis; dcSSc, diffuse cutaneous systemic sclerosis; SIR, standard incidence ratio; CI, confidence interval; FT4, free thyroxine; FT3, free triiodothyronine; anti-TPO, anti-thyroid peroxidase; anti-Tg, anti-thyroglobulin; TSH, thyroid-stimulating hormone; AITDs, autoimmune thyroid diseases; HT, Hashimoto’s thyroiditis; GD, Graves’ disease; SD, standard deviation; PTC, papillary thyroid cancer; ACAs, anti-centromere antibodies; NS, not significant; EG, euthyroid goiter.

**Table 2:** Thyroid volume and nodules in systemic sclerosis patients.

Study, Year, Reference	Design	Patients	Study Findings
Badak SO 2022 [71]	Cross-sectional study	N = 86 P with SSc N1 = 40 (46.5%) P with non-decreased thyroid volume N2 = 46 (53.5%) P with atrophic thyroid volume	Age (years), median ± std: N1 = 48.98 ± 8.850; N2 = 56.02 ± 10.23; [*p*-value = 0.001] Duration of SSc (months), median (min–max): N1 = 48 (5–300); N2 = 256 (24–480); [*p*-value ≤ 0.001] MSS, median (min–max): N1 = 4 (2–13); N2 = 10 (3–24); [*p*-value ≤ 0.001] mRSS, median (min–max): N1 = 10 (2–36); N2 = 34 (1–43); [*p*-value ≤ 0.001] TSH (mlU/L), median (min–max): N1 = 5 (2–11); N2 = 3 (0.1–5.2); [*p*-value ≤ 0.001] Sex: Females N1 = 36 P (46.1); N2 = 42 P (53.8%). Males N1 = 4 P (50%); N2 = 4 P (50%); [*p*-value ≥ 0.05] Immunosuppressive: Negative: N1 = 25 P (53.3%); N2 = 21 P (44.7%) Positive: N1 = 15 P (36.6%); N2 = 25 P (63.4%) [*p*-value ≥ 0.05] Smoking: Negative: N1 = 36 (46.2%); N2 = 42 P (53.8%) Positive: N1 = 4 P (50%); N2 = 4 P (50%) [*p*-value ≥ 0.05] RP: Negative: N1 = 2 P (66.7%); N2 = 1 P (33.3%) Positive: N1 = 38 P (45.9%); N2 = 45 P (54.1%) [*p*-value ≤ 0.001] ILD: Negative: N1 = 25 P (74.3%); N2 = 9 P (25.7%) Positive: N1 = 15 P (28.3%); N2 = 37 P (71.7%) [*p*-value ≤ 0.001] PAH: Negative: N1 = 36 P (52.1%); N2 = 37 P (47.9%) Positive: N1 = 4 P (23.5%); N2 = 13 P (76.5%) [*p*-value ≤ 0.001] Gastrointestinal involvement: Negative: N1 = 18 P (66.7%); N2 = 10 P (33.3%) Positive: N1 = 20 P (36.2%); N2 = 36 P (63.8%) [*p*-value = 0.007] Thyroid autoantibody: Negative: N1 = 32 P (43.4%); N2 = 42 P (56.6%) Positive: N1 = 8 P (66.7%); N2 = 4 P (33.3%) [*p*-value ≥ 0.05] ANAs: Negative: N1 = 2 P (55.6%); N2 = 1 P (33.3%) Positive: N1 = 38 P (45.9%); N2 = 45 P (54.1%) [*p*-value ≥ 0.05] SCL 70 Ab: Negative: N1 = 20 P (55.6%); N2 = 16 P (44.4%) Positive: N1 = 20 P (40.4%); N2 = 30 P (59.6%) [*p*-value ≥ 0.05] ACAs: Negative: N1 = 25 P (40.6%); N2 = 37 P (59.4%) Positive: N1 = 15 P (64.5%); N2 = 9 P (37.5%) [*p*-value ≥ 0.05] SSc subtype: Limited: N1 = 20 P (64.5); N2 = 10 P (35.5%) Diffuse: N1 = 20 P (36.8%); N2 = 36 P (63.2%) [*p*-value = 0.013]
Meridor K 2022 [65]	Prospective study	N = 50 P with SSc N1 = 10 (20%) P with thyroid disease previously diagnosed N2 = 40 (80%) P not previously diagnosed with thyroid disease N3 = 40 (80%) P with diffuse SSc N4 = 10 (20%) P with limited SSc	Normal thyroid size: N = 38 P (76%), N1 = 6 P (60%); N2 = 32 P (80%) Enlarged thyroid size: N = 8 P (16%); N1 = 1 P (10%); N2 = 7 P (17.5%) Reduced thyroid size: N = 4 P (8%); N1 = 3 P (30%); N2 = 1 P (2.5%) Thyroid nodules: N = 22 P (44%); N1 = 3 P (30%); N2 = 19 P (47.5%); [*p*-value = 0.480] Thyroid nodules > 1 cm: N = 12 P (24%); N1 = 2 P (20%); N2 = 10 P (25%); [*p*-value = 1.000] Thyroid nodules for FNA: N = 6 P (12%); N1 = 2 P (20%); N2 = 4 P (10%) [*p*-value = 0.586]
Antonelli A 2016 [58]	Cross-sectional study	N = 1635 P N1 = 327 P with SSc N2 = 654 P in the control group from an iodine-deficient area N3 = 654 P in the control group from an iodine-sufficient area	Thyroid nodules, %: N1 = 28 (91/236); N2 = 25 (163/491); N3 = 15 (98/556); [*p*-value < 0.0001]
Toki S 2014 [59]	Observational retrospective study	N = 210 P with SSc N1 = 30 P with AITDs N2 = 180 P without AITDs	Ultrasonography findings: Thyroid volume > 20 mL (%): N = 6.3 (4/63); N1 = 6.7 (2/30); N2 = 6.1 (2/33); [*p*-value = 0.922] Thyroid volume < 6 mL (%): N = 6.3 (4/63); N1 = 13.3 (4/30); N2 = 0; [*p*-value < 0.05] Thyroid nodule (%): N = 19 (12/63); N1 = 16.7 (5/30); N2 = 21.2 (7/33); [*p*-value = 0.646]
Antonelli A 2013 [62]	Longitudinal study	N = 358 P N1 = 179 P with SSc N2 = 179 P without SSc in control group	Initial thyroid status: Thyroid volume, ml (SD): N1 = 11 (10); N2 = 14 (13); [*p*-value = 0.044] Thyroid volume > 20 mL, %: N1 = 11; N2 = 12; [*p*-value = NS] Thyroid volume < 6 mL, %: N1 = 12; N2 = 5; [*p*-value = 0.015] Thyroid nodules, %: N1 = 39; N2 = 43; [*p*-value = NS] Last evaluation: Hypoechoic pattern, %: N1 = 32; N2 = 15; [*p*-value < 0.0001] Thyroid volume, ml (SD): N1 = 10 (11); N2 = 14 (12); [*p*-value = 0.037] Thyroid volume > 20 mL, %: N1 = 11; N2 = 14; [*p*-value = NS] Thyroid volume < 6 mL, %: N1 = 16; N2 = 6; [*p*-value = 0.004] Thyroid nodules, %: N1 = 44; N2 = 50; [*p*-value = NS] New cases: Hypoechoic pattern: N1 = 16 (14.6); N2 = 5 (3.5); [*p*-value = 0.013] Thyroid volume > 20 mL: N1 = 0 (0); N2 = 2 (1.4); [*p*-value = NS] Thyroid volume < 6 mL: N1 = 6 (5.4); N2 = 2 (1.4); [*p*-value = NS] Thyroid nodules: N1 = 9 (8.2); N2 = 12 (8.5); [*p*-value = NS] Thyroid nodules: N1 = 9 (8.2); N2 = 12 (8.5); [*p*-value = NS]
Antonelli A 2007 [64]	Cross-sectional study	N = 606 P N1 = 202 P with SSc N2 = 404 P controls N3 = 184 P females with SSc N4 = 18 P males with SSc N5 = 368 P female controls N6 = 36 P male controls	Female patients: Thyroid volume (ml): N3 = 10 ± 9; N5 = 13 ± 14; [*p*-value = 0.0156]. Thyroid volume > 20 mL: N3 = 21/163 (11%); N5 = 44/324 (12%); [*p*-value = NS]. Thyroid volume < 6 mL: N3 = 54/130 (29%); N5 = 70/298 (19%); [*p*-value = 0.008]. Thyroid nodules: N3 = 96/88 (52%); N5 = 210/158 (57%); [*p*-value = NS]. Male patients: Thyroid volume (ml): N4 = 14 ± 5; N6 = 18 ± 12; [*p*-value = NS]. Thyroid volume > 20 mL: N4 = 3/15 (17%); N6 = 8/28 (22%); [*p*-value = NS]. Thyroid volume < 6 mL: N4 = 0; N6 = 0; [*p*-value = NS]. Thyroid nodules: N4 = 6/12 (33%); N6 = 13/23 (36%); [*p*-value = NS];

Abbreviations: P, patients; SSc, systemic sclerosis; ACAs, anti-centromere antibodies; ANAs, antinuclear antibodies; mRSS, modified Rodnan skin score; RP, Raynaud’s phenomenon; ILD, interstitial lung disease; PAH, pulmonary arterial hypertension; NS, not significant; TSH, thyroid-stimulating hormone.

**Table 3:** Clinical manifestations in systemic sclerosis patients with or without thyroid disorders.

Study, Year, Reference	Design	Patients	Study Findings
Paolee Y 2021 [72]	Observational study	N = 200 P with SSc N1 = 166 P with normal thyroid hormone N2 = 31 P with clinical or subclinical hypothyroidism	Age at onset > 60 years: N1 = 26 P (15.7%); N2 = 6 P (19.4%); [*p*-value = 0.60]; OR = 1.30 (0.40–3.60); Duration of disease < = 4 years: N1 = 76 P (45.8%); N2 = 13 P (41.9%); [*p*-value = 0.84]; OR = 0.86 (0.36–2.00); Female: N1 = 107 P (64.5%); N2 = 25 P (80.6%); [*p*-value = 0.09]; OR = 2.30 (0.85–7.21); dcSSc subtype: N1 = 115 (69.3%); N2 = 22 P (70.9%); [*p*-value = 1.00]; OR = 1.08 (0.45–2.86); BMI < 18.5 kg/m^2^: N1 = 14 P (29.2%); N2 = 2 P (28.5%); [*p*-value = 1.00]; OR = 0.97 (0.08–6.84); mRSS > 20 points: N1 = 17 P (10.2%); N2 = 4 (12.9%); [*p*-value = 0.75]; OR = 1.30 (0.30–4.41); RP: N1 = 103 P (62.2%); N2 = 9 (29.0%); [*p*-value = 0.001]; crude OR = 0.25 (0.09–0.61); adjusted OR = 0.28 (0.11–0.66); [*p*-value = 0.004] Digital ulcer: N1 = 41 P (24.7%); N2 = 5 P (16.1%); [*p*-value = 0.36]; OR = 0.61 (0.17–1.79); Digital gangrene: N1 = 1 P (0.6%); N2 = 0 P; OR = NA; Telangiectasia: N1 = 36 P (21.7%); N2 = 9 P (29.0%) [*p*-value = 0.36]; OR = 1.5 (0.56–3.78); Calcinosis cutis: N1 = 5 P (3.1%); N2 = 2 P (6.4%); [*p*-value = 0.30]; OR = 2.19 (0.20–14.10); Salt and pepper skin: 66 P (40.2%); N2 = 12 P (38.7%); [*p*-value = 1.00]; OR = 0.93 (0.38–2.19); Edematous skin: N1 = 27 P (16.5%); N2 = 7 P (22.5%); [*p*-value = 0.44]; OR = 1.48 (0.49–1.98); Tendon friction rubs: N1 = 22 P (13.4%); N2 = 2 P (6.4%); [*p*-value = 0.38]; OR = 0.44 (0.04–1.98); Hand deformity: N1 = 50 P (30.5%); N2 = 10 P (32.2%); [*p*-value = 0.83]; OR = 1.08 (0.42–2.62); Arthritis: N1 = 14 P (8.5%); N2 = 2 P (6.4%); [*p*-value = 1.00]; OR = 0.73 (0.07–3.50); Muscle weakness: N1 = 14 P (8.5%); N2 = 0 P; OR = NA; Esophageal involvement: N1 = 58 P (35.4%); N2 = 13 P (41.9%); [*p*-value = 0.80]; OR = 1.32 (0.55–3.08); Stomach involvement: N1 = 29 P (17.7%); N2 = 6 P (19.3%); [*p*-value = 0.80]; OR = 1.11 (0.34–3.13); Constipation: N1 = 30 P (18.3%); N2 = 5 P (16.1%); [*p*-value = 1.00]; OR = 0.85 (0.23–2.53); Weight loss: N1 = 45 P (27.4%); N2 = 3 P (9.6%); [*p*-value = 0.04]; crude OR = 0.28 (0.05–0.99); adjusted OR = 0.31 (0.08–1.10); [*p*-value = 0.07] Pulmonary fibrosis: N1 = 66 P (39.7%); N2 = 8 P (25.8%); [*p*-value = 0.16]; crude OR = 0.52 (0.19–1.31); PAH: N1 = 14 (8.4%); N2 = 0 P; OR = NA; Anemia: N1 = 76 P (45.7%); N2 = 21 P (67.7%) [*p*-value = 0.03]; crude OR = 2.48 (1.03–6.27); adjusted OR = 2.74 (1.17–6.47); [*p*-value = 0.02]; FVC < 70%: N1 = 48 P (55.1%); N2 = 5 P (71.4%); [*p*-value = 0.46]; OR = 2.03 (0.30–22.20); Pericardial effusion present: N1 = 14 P (14.4%); N2 = 2 P (14.2%); [*p*-value = 1.00]; OR = 0.98 (0.09–5.22); LVEF < 50%: N1 = 4 (4%); N2 = 0 P; OR = NA
Triggianese P 2017 [73]	Case-control study	N = 77 P pregnant with SSc N1 = 41 P with thyroid disorders N2 = 36 P without thyroid disorders N3 = 23 P with pregnancy failure N4 = 54 P without pregnancy failure	Limited SSc: N = 44 P; N1 = 33 P (80%); N2 = 21 P (58.4%); [*p*-value < 0.05] Diffuse SSc: N = 23 P; N1 = 8 P (19.5%); N2 = 15 P (41.6%); [*p*-value < 0.05] Sicca syndrome: N = 15 P; N1 = 8 P (19.5%); N2 = 7 P (19.5%) [*p*-value > 0.05] ANAs: N = 61 P; N1 = 31 P (75.6%); N2 = 30 P (83.4%); [*p*-value > 0.05] Digital ulcers: N = 18 P; N1 = 5 P (12.2%); N2 = 13 P (36.2%); [*p*-value < 0.05] Heart: elevated US-PAPs (*n*/%): N = 22 P; N1 = 9 P (22%); N2 = 12 P (36.2%); [*p*-value > 0.05] Lung: reduced DLCO (*n*/%): N = 32 P; N1 = 17 P (41.5%); N2 = 15 P (41.7%); [*p*-value > 0.05] Esophageal reflux, dysphagia (n/%): N = 33 P; N1 = 12 P (29.3%); N2 = 21 P (58.4%); [*p*-value < 0.05] Women with PF (*n*/%): N = 23 P; N1 = 13 P (31.7%); N2 = 10 P (27.8%); [*p*-value > 0.05] RSA (n/%): N1 = 6 P (46.2%); N2 = 3 P (30%); [*p*-value > 0.05] Primary infertility (*n*/%): N1 = 2 P (15.4%); N2 = 1 P (10%); [*p*-value > 0.05] PD (n/%): N1 = 2 P (15.4%); N2 = 3 P (30%); [*p*-value > 0.05] IUGR (n/%): N1 = 3 P (23%); N2 = 1 P (10%); [*p*-value > 0.05] IFD (n/%): N1 = 0 P; N2 = 2 P (20%); [*p*-value > 0.05] HT (n/%): N3 = 9/62; N4 = 10/35.7; [*p*-value < 0.05] dcSSc (n/%): N3 = 11/47.8; N4 = 12/22.2; [*p*-value < 0.05] lcSSc (n/%): N3 = 12/52.2; N4 = 42/77.8; [*p*-value < 0.05]
Posselt RT 2017 [57]	Cross-sectional study	N = 58 P with SSc N1 = SSc patients with anti-TPO and hypothyroidism N2 = SSc patients with anti-thyroid antibodies without hypothyroidism N3 = SSc patients without anti-thyroid antibodies	mRSS: N = 15.0 ± 10.1; N1 = 11.1 ± 9.8; N2 = 15.3 ± 7.4; N3 = 15.8 ± 10.8; [*p*-value = 0.70] Medsger index (median [IQR]): N = 5.0 [4.0–7.7]; N1 = 5.5 [3.0–9.0]; N2 = 4.0 [3.0–7.0]; N3 = 6.0 [4.0–8.0]; [*p*-value = 0.43]
Wielosz E 2016 [66]	Cross-sectional study	N = 86 P with SSc N1 = 32 P with diffuse SSc N2 = 54 P with limited SSc N3 = 24 P with SSc and anti-TPO/Anti-Tg-positive N4 = 59 P with SSc and anti-TPO/Anti-Tg-negative	Anti-TPO: N = 22 P (26%); N1 = 8 P (25%); N2 = 14 P (26%); [*p*-value = NS] Anti-Tg: N = 22 P (26%); N1 = 9 P (28%); N2 = 13 P (24%); [*p*-value = NS] Anti-TPO or Anti-Tg: N = 27 P (31%); N1 = 11 P (34%); N2 = 16 P (30%); [*p*-value = NS] Anti-TPO and Anti-Tg: N = 17 P (20%); N1 = 6 P (19%); N2 = 11 P (20%); [*p*-value = NS] AITDs: N = 26 P (30%); N1 = 10 P (31%); N2 = 16 P (30%); [*p*-value = NS] Calcinosis: N3 = 8 P (30%); N4 = 13 P (22%); [*p*-value = NS] Digital ulcers: N3 = 4 P (15%); N4 = 11 P (19%); [*p*-value = NS] Gastrointestinal tract involvement: N3 = 18 P (67%); N4 = 37 P (63%); [*p*-value = NS] ILD: N3 = 16 P (53%); N4 = 26 P (44%); [*p*-value = NS] Primary heart involvement: N3 = 9 P (33%); N4 = 22 P (37%); [*p*-value = NS] PAH (echocardiography): N3 = 6 P (22%); N4 = 11 P (19%); [*p*-value = NS] Kidney involvement: N3 = 7 P (25%); N4 = 16 P (27.1%); [*p*-value = NS] SRC: N3 = 0 P; N4 = 2 P (3%); [*p*-value = NS] Joint involvement arthralgia: N3 = 23 P (85%); N4 = 48 P (81%); [*p*-value = NS] Arthritis: N3 = 10 P (37%); N4 = 21 P (36%); [*p*-value = NS] Overlap syndromes: N3 = 7 P (25%); N4 = 10 P (16%); [*p*-value = NS] Myalgia or myositis: N3 = 5 P (19%); N4 = 12 P (20%); [*p*-value = NS] Anti-Scl-70 antibodies: N3 = 11 P (41%); N4 = 18 P (33%); [*p*-value = NS] ACAs: N3 = 6 P (22%); N4 = 13 P (16%); [*p*-value = NS]
Costa CC 2014 [74]	Cross-sectional study	N = 56 P with SSc N1 = 11 P with HT N2 = 45 P without HT	Ethnicity: Caucasian: N1 = 63.6% (7/11); N2 = 65.9% (29/44); African descent: N1 = 36.3% (4/11); N2 = 31.82% (14/44); Indian descent: N2 = 2.28% (1/44) [*p*-value = 0.31] Gender Female/Male: N1 = 10/1; N2 = 42/3; [*p*-value = 1.0] Median age of patient: N1 = 56 (IQI = 25–69); N2 = 54 (IQI = 19–81); [*p*-value = 0.87] Form of scleroderma: Diffuse: N1 = 27.3% (3/11); N2 = 35.5% (16/45); Limited: N1 = 72.7% (8/11); N2 = 64.4% (29/45); [*p*-value = 1.0] [*p*-value = 0.18]
Toki S 2014 [59]	Observational retrospective study	N = 210 P with SSc N1 = 30 P with AITDs N2 = 180 P without AITDs	GD (%): N = 0.48 (1/210); N1 = 3.3 (1/30); HT (%): N = 13.8 (29/210); N1 = 96.7 (29/30); Clinical hypothyroidism (%): N = 9.5 (20/210); N1 = 66.7 (20/30); Subclinical hypothyroidism (%): N = 4.3 (9/210); N1 = 30.0 (9/30); Anti-Tg (%): N = 19.5 (41/210); N1 = 66.7 (20/30); N2 = 14.3 (21/180); Anti-TPO (%): N = 22.4 (47/210); N1 = 73.3 (22/30); N2 = 14.8 (25/180); Anti-Tg or Anti-TPO (%): N = 28.6 (60/210); N1 = 100 (30/30); N2 = 16.7 (30/180); Type: localized SSc (%): N = 70.5 (148/210); N1 = 80.0 (24/30); N2 = 68.9 (124/180); diffuse SSc (%): N = 29.5 (62/210); N1 = 20.0 (6/30); N2 = 31.1 (56/180) [*p*-value = 0.217] Autoantibody: ANA (%): N = 90.0 (189/210); N1 = 100 (30/30); N2 = 88.3 (159/180) [*p*-value < 0.05] Topo I (%): N = 21.0 (44/210); N1 = 6.7 (2/30); N2 = 22.3 (42/180) [*p*-value < 0.005] RNP (%): N = 12.9 (27/210); N1 = 16.7 (5/30); N2 = 12.2 (22/180) [*p*-value = 0.501] ACA (%): N = 43.3 (91/210); N1 = 63.3 (19/30); N2 = 40.0 (72/180) [*p*-value < 0.05] SS-A (%): N = 26.2 (55/210); N1 = 36.7 (11/30); N2 = 24.4 (44/180) [*p*-value = 0.159] SS-B (%): N = 4.3 (9/210); N1 = 3.3 (1/30); N2 = 4.4 (8/180) [*p*-value = 0.781] Complications: ILD (%): N = 41.0 (86/210); N1 = 30.0 (9/30); N2 = 42.8 (77/180) [*p*-value = 0.188] PAH (%): N = 8.1 (17/210); N1 = 10 (3/30); N2 = 7.8 (14/180) [*p*-value = 0.68] DU (%): N = 17.1 (36/210); N1 = 16.7 (5/30); N2 = 17.2 (31/180) [*p*-value = 0.94] CI (%): N = 10.0 (21/210); N1 = 13.3 (4/30); N2 = 9.4 (17/180) [*p*-value = 0.511] SjS (%): N = 30.0 (63/210); N1 = 50.0 (15/30); N2 = 26.7 (48/180) [*p*-value < 0.001] Ultrasonography findings: Thyroid volume > 20 mL (%): N = 6.3 (4/63); N1 = 6.7 (2/30); N2 = 6.1 (2/33); [*p*-value = 0.922] Thyroid volume < 6 mL (%): N = 6.3 (4/63); N1 = 13.3 (4/30); N2 = 0; [*p*-value < 0.05] Thyroid nodule (%): N = 19 (12/63); N1 = 16.7 (5/30); N2 = 21.2 (7/33); [*p*-value = 0.646] Thyroid cancer (%): N = 1.7 (1/63); N1 = 0 (0/30); N1 = 3 (1/33); [*p*-value = 0.345]
Bagnato 2015 [67]	Cross-sectional study	N = 105 P N1 = 70 P with SSc N2 = 35 P controls with HT N3 = 30 P with diffuse cutaneous SSc N4 = 40 P with limited cutaneous SSc	Age (year): N1 = 47 ± 11; N2 = 38 ± 16; N3 = 47 ± 10; N4 = 46 ± 10; [*p*-value = 0.0027] RP duration: N1 = 12 ± 8; N2 = /; N3 = 12 ± 9; N4 = 12 ± 8; [*p*-value = /] mRSS: N1 = 14 ± 9; N2 = 4 ± 6; N3 = 20 ± 8; N4 = 9 ± 6; [*p*-value < 0.0001] ANA positive (%): N1 = 68 (97); N2 = 0; N3 = 30 (100); N4 = 38 (85); [*p*-value < 0.0001] SCL70 Ab positive (%): N1 = 31 (44); N2 = 0; N3 = 26 (86); N4 = 5 (12); [*p*-value < 0.0001] ACA positive (%): N1 = 19 (27); N2 = 0; N3 = 5 (16); N4 = 14 (35); [*p*-value < 0.0001]
Avouac J 2010 [68]	Observational study	N = 1132 P with SSc N1 = 585 P with SSc from France N2 = 547 P with SSc from Italy N3 = 70 P with SSc and autoimmune thyroiditis N4 = 1062 P with SSc without autoimmune thyroiditis	Autoimmune thyroiditis [n (%)]: N = 70 (6); N1 = 23 (4); N2 = 47 (8.5); [*p*-value = 0.025] Female, n (%): N3 = 68/70 (97%); N4 = 921/1062 (87%); [*p*-value = 0.04] Age, mean ± SD, yrs: N3 = 62 ± 13; N4 = 63 ± 13; [*p*-value = 0.9] Disease duration, yrs: N3 = 11 ± 9; N4 = 12 ± 9; [*p*-value = 0.5] lcSSc, n (%): N3 = 55/70 (78); N4 = 750/1062 (71); [*p*-value = 0.2] History of digital ulcers, n (%); N3 = 15/70 (22); N4 = 402/1062 (38); [*p*-value = 0.03] PAH, n (%): N3 = 7/70 (10); N4 = 112/1062 (10); [*p*-value = 0.25] Pulmonary fibrosis on CT scan, n (%): N3 = 13/70 (18); N4 = 382/1062 (36); [*p*-value = 0.001]; OR = 0.3 (0.1–0.7): [*p* = 0.004]; ANAs (>1/160), n (%): N3 = 66/70 (94); N4 = 980/1062 (92); [*p*-value = 0.06] ATAs, n (%): N3 = 14/70 (20); N4 = 271/1062 (25); [*p*-value = 0.4] ACAs, n (%): N3 = 41/70 (58); N4 = 428/1062 (40); [*p*-value = 0.02]; Decreased forced vital capacity, n (%): N3 = 8/70 (11); N4 = 158/1062 (15); [*p*-value = 0.3]; Decreased DLCO/AV, n (%): N3 = 27/70 (39); N4 = 446/1062; [*p*-value = 0.3]; Immunosuppressive drug, n (%): N3 = 7/70 (10); N4 = 265/1062 (25); [*p*-value = 0.003]
Ugurlu S 2008 [75]	Case-control study	N = 50 P female N1 = 24 P with autoimmune hypothyroidism N2 = 26 P controls	FT4: N1 = 1.07 ± 0.18 ng/dL; N2 = 1.10 ± 0.19; TSH: N1 = 10.80 ± 7.78 mIU/mL; N2 = 1.44 ± 0.78 MIU/mL; [*p*-value < 0.001]; Anti-TPO (median [25th, 75th percentiles]): N1 = 567 [79.3,100] IU/mL; N2 = 0.13 [0.01,1.59] IU/mL; [*p* < 0.001]; Anti-Tg (median [25th, 75th percentiles]): N1 = 103 [20.9,1000] IU/mL; N2 = 0.78 [0.53,20] UI/mL; [*p*-value < 0.001]; Anti-Scl-70 index values: N1 = 0.48 ± 0.12; N2 = 0.37 ± 0.13; [*p* = 0.002]

Abbreviations: P, patients; SSc, systemic sclerosis; ACAs, anti-centromere antibodies; ATAs, anti-topoisomerase I antibodies; ANAs, antinuclear antibodies; mRSS, modified Rodnan skin score; RP, Raynaud’s phenomenon; ILD, interstitial lung disease; PAH, pulmonary arterial hypertension; NS, not significant; TSH, thyroid-stimulating hormone; lcSSc, limited cutaneous systemic sclerosis; dcSSc, diffuse cutaneous systemic sclerosis; FT4, free thyroxine; anti-TPO, anti-thyroid peroxidase; anti-Tg, anti-thyroglobulin; AITDs, autoimmune thyroid diseases; HT, Hashimoto’s thyroiditis; GD, Graves’ disease; IUGR, intrauterine growth restrictions; RSA, recurrent spontaneous abortions; PF, pregnancy failure; BMI, body mass index; OR, odds ratio; FVC, forced vital capacity; DLCO, diffusing capacity for carbon monoxide; DLCO/AV, diffusing capacity for carbon monoxide/alveolar volume; LVEF, left ventricular ejection fraction; CT, computer tomography; US-PAPs, systolic pulmonary arterial pressure on heart ultrasound; PD, preterm delivery; IFD, intrauterine fetal death.

**Table 4:** Thyroid autoimmunity in relatives of systemic sclerosis patients.

Study, Year, Reference	Design	Patients	Study Findings
Meridor K 2022 [65]	Prospective study	N = 50 P with SSc N1 = 10 (20%) P with thyroid disease previously diagnosed N2 = 40 (80%) P not previously diagnosed with thyroid disease N3 = 40 (80%) P with dcSSc N4 = 10 (20%) P with lcSSc	Thyroid autoimmune disease in first-degree relatives N = 16 P (33%) P (16%); N1 = 4 P (40%); N2 = 12 P (31%); [*p*-value = 0.420]
Antonelli A 2016 [58]	Cross-sectional study	N = 1635 P N1 = 327 P with SSc N2 = 654 P in the control group from an iodine-deficient area N3 = 654 P from an iodine-sufficient area N4 = 321 P with SSc without PTC cancer N5 = 6 P with SSc and PTC	Familial thyroid disease, %: N1 = 38; N2 = 46; N3 = 18. [*p*-value < 0.0001]
Koumakis E 2012 [78]	Cross-sectional study	N = 623 families N1 = 373 families of the SSc patients N2 = 250 control families N3 = 823 first-degree relatives of SSc patients N4 = 318 first-degree relatives of controls	Female sex, *n* (%): N1 = 327 (87.7%); N2 = 211 (84.4); [*p*-value = 0.27] Mean age, yrs (SD): N1 = 61.1(13.0); N2 = 57.5 (13.4); [*p*-value = 0.98] No. families with at least one autoimmune disease (%): N1 = 121 (32.4); N2 = 49 (19.6); [*p*-value = 0.0006] No. FDR (SD): N1 = 823; N2 = 318. Mean no. FDR (SD): N1 = 6.8 (2.9); N2 = 6.5 (2.5); [*p*-value = 0.18] Mean no. offspring (SD): N1 = 1.8 (1.3); N2 = 2.1 (1.1); [*p*-value = 0.10] Mean no. siblings (SD): N1 = 3.0 (2.3); N2 = 2.4 (2.1); [*p*-value = 0.08] No. FDR with available medical history (%): N1 = 679 (82.5); N2 = 271 (85.0); [*p*-value = 0.35] Concomitant autoimmune disease (%): > = 1 N1 = 164 (44.0) > = 2 N1 = 35 (9.4) > = 3 N1 = 9 (2.4) AITDs: N1 = 49 (13.1) Disease in first-degree relatives, n (%): AITDs: N3 = 40 (4.9); N4 = 5 (1.6); [*p*-value = 0.01] [λR = 3.1 OR (95% CI) = 3.20 (1.25–8.180] HT: N1 = 26 (2.8); N2 = 3 (0.9); [*p*-value = 0.04 λR = 3.0] GD: N1 = 11 (1.9); N2 = 2 (0.6); [*p*-value = 0.18 λR = 3.1]

Abbreviations: P, patients; SSc, systemic sclerosis; lcSSc, limited cutaneous systemic sclerosis; dcSSc, diffuse cutaneous systemic sclerosis; CI, confidence interval; AITDs, autoimmune thyroid diseases; HT, Hashimoto’s thyroiditis; GD, Graves’ disease; SD, standard deviation; FDR, first-degree relatives.
